# Topology predicts long-term functional outcome in early psychosis

**DOI:** 10.1038/s41380-020-0826-1

**Published:** 2020-07-06

**Authors:** Margot Fournier, Martina Scolamiero, Mehdi M. Gholam-Rezaee, Martine Cleusix, Raoul Jenni, Carina Ferrari, Philippe Golay, Philipp S. Baumann, Michel Cuenod, Philippe Conus, Kim Q. Do, Kathryn Hess

**Affiliations:** 1 Center for Psychiatric Neuroscience, Department of Psychiatry, Lausanne University Hospital and University of Lausanne, CHUV—UNIL, Lausanne, Switzerland; 2 SV BMI UPHESS, École Polytechnique Fédérale de Lausanne, CH-1015 Lausanne, Switzerland; 3 Service of General Psychiatry, Department of Psychiatry, Lausanne University Hospital and University of Lausanne, CHUV—UNIL, Lausanne, Switzerland; 4 Present Address: Mathematics Department, KTH Royal Institute of Technology, Stockholm, Sweden

**Keywords:** Schizophrenia, Predictive markers

## Abstract

Early intervention in psychosis is crucial to improving patient response to treatment and the functional deficits that critically affect their long-term quality of life. Stratification tools are needed to personalize functional deficit prevention strategies at an early stage. In the present study, we applied topological tools to analyze symptoms of early psychosis patients, and detected a clear stratification of the cohort into three groups. One of the groups had a significantly better psychosocial outcome than the others after a 3-year clinical follow-up. This group was characterized by a metabolic profile indicative of an activated antioxidant response, while that of the groups with poorer outcome was indicative of oxidative stress. We replicated in a second cohort the finding that the three distinct clinical profiles at baseline were associated with distinct outcomes at follow-up, thus validating the predictive value of this new stratification. This approach could assist in personalizing treatment strategies.

## Introduction

Early intervention in psychosis is key to improving long-term outcome [1–6], which is strongly influenced by quality of life and social functioning trajectories in the early treatment phase [7, 8]. Clinical and biological markers quantifying disease course or treatment response should therefore be developed to guide treatment decision at early stages of the disease [9–12]. Since patient heterogeneity hampers marker identification, stratification tools allowing personalized functional-disability preventive strategies are needed.

Topological Data Analysis (TDA) is a powerful approach to analyzing the shape of biological datasets, identifying biotypes in a data-driven way. The TDA algorithm Mapper produces graphical output that allows insight into the robustness of the proposed stratification [13]. Mapper has shown promise for outcome assessment in neurological trauma [14] and for detecting significant subgroups of breast cancers. Applied to fragile X-syndrome [15], structural brain images, Mapper identified distinct subgroups with consistent cognition, adaptive functioning, and autism severity scores.

In this study, we used TDA to stratify a cohort of early psychosis patients based on Positive and Negative Syndrome Scale (PANSS) scores. Although clustering algorithms were used previously to study symptom patterns [16], this is the first such application of TDA. TDA stratified the early psychosis cohort into three groups with coherent clinical profiles. We then sought a biosignature of the groups, representing distinct underlying pathophysiological pathways. To test the hypothesis of redox dysregulation/oxidative stress as one pathophysiological hub [17–20], we studied redox markers such as blood levels of glutathione (GSH), GSH peroxidase and reductase activities (GPx, GR), thioredoxin (Trx), and GSH-related metabolites. In addition, we explored an unbiased selection of 28 amino acids of which metabolism is notably altered both at schizophrenia onset and in later stages [21–25].

To validate the clinical relevance of our stratification, we compared outcomes after 3 years between groups, focusing on functional level for three reasons. First, functional recovery, the main determinant of the social and economic burdens of schizophrenia [7], may fail despite symptom remission. Second, functional level in the early illness phase is relatively stable over 20 years [7] and the strongest predictor of later functional level [26]. The 3-year follow-up in our study is thus likely to predict longer-term outcomes. Finally, functional impairment, reported in all forms of psychosis [7], is an important indicator for individuals at risk for psychosis [27]. Better knowledge of its determinants is therefore useful at various stages of the illness.

## Materials/subjects and methods

### Participants

All subjects provided a fully informed written consent; all procedures were in accordance with the ethical standards of the Helsinki Declaration (1983) and approved by the local ethical committee. EPP were recruited from the Treatment and early Intervention in Psychosis Program (TIPP^2^, see Supplementary). Cohort 1 comprises 101 patients from the TIPP program (biomarkers project, globally representative of the entire cohort [28]) and cohort 2 comprises 93 TIPP patients (not included in the biomarker study).

### Clinical evaluations

Psychopathological assessments were conducted by trained psychologists. We used the PANSS and the Wallwork five-factor model to assess symptom severity and to categorize the different symptom domains [29].

Outcomes at discharge were evaluated at 36 months or within the last year of treatment: (i) ‘Working’: Modified Vocational Status Index [30]; (ii) ‘Living independently’: Modified Location Code Index [30]; (iii) symptomatic remission: Andreasen criteria [31]; (iv) Social and Occupational Functioning Assessment Scale (composite global score; SOFAS) [32]; (v) Global Assessment of Functioning (global composite score; GAF) [32].

### TDA stratification of cohort 1 with Mapper algorithm

We used the Mapper algorithm [33] (www.ayasdi.com) to visualize and study the 30 PANSS items at baseline, using (i) distance: normalized Pearson correlation distance; (ii) filter function: coefficients in the coordinate system given by the first two principal components; (iii) resolution: 60, (iv) gain: 7. Our choice of resolution and gain is not crucial for the identification of the Mapper-based partition [A, B, C], as these groups are easily recognizable whenever the Mapper graph does not have trivial connectivity structure (Supplementary Fig. 1, Supplementary Methods).

### Assessment of metabolic markers

Amino acids were quantified in plasma [34], and GSH and GPx, GR, and Trx activity levels in blood cell pellets after hemolysis [35–37] (Supplementary Methods). Two metabolites are considered strongly correlated in a group of patients if the Pearson correlation between the associated vectors is significantly different from zero (*p* value < 0.05). Biological correlations among the 28 measured metabolites in cohort 1 were determined in groups A, B, or C as detailed in the Supplementary.

### Validation of the stratification in cohort 2

An independent cohort of 93 individuals, called cohort 2, was used for validation. We computed the centroid of the symptom vectors of each group in cohort 1, i.e., the average value, within the group, of each coordinate in the 30-dimensional space of PANSS scores. We then computed the Euclidean distance between the symptom vector of each patient in cohort 2 and the centroids of the groups from cohort 1, assigning the patient to the group with the closest centroid. This assignment determined a partition of cohort 2 into subgroups, called the replicated groups.

### Prediction of good vs. poor functional outcome

We used logistic regression to predict good vs. poor functional outcome (GAF > 65 vs. GAF ≤ 65), based on either the PANSS items or group membership in [A, B, C] or [0, 1, 2]. We trained on cohort 1, validated on cohort 2, and evaluated the accuracy, precision, and recall of the regression, as implemented in the sklearn library. See the Supplementary and Supplementary Table 1 for more details.

### Group comparisons for clinical and metabolic data

We used the Kolmogorov–Smirnov test, as implemented in the python function ‘scipy.stats.ks_2samp’ from the scipy library, to identify which PANSS items are group-specific. For each PANSS item, we compared the distribution of its scores in one group with the distribution in the remaining two groups by the Kolmogorov–Smirnov test and applied Bonferroni correction for multiple comparisons. Items were considered as group-specific if the corrected *p* value < 0.05.

We computed Pearson correlation coefficients to create correlation matrices of metabolite levels in patients within groups A, B, or C, using the function *corrcoef()*, in the numpy python library. All other statistics were performed with R, using the Chi-Square test for independence to compare distributions and Student’s *t* test or analysis of variance to compare continuous variables between two or three groups, respectively. If not otherwise specified, groups were considered different when *p* < 0.05.

### k-means clustering

We performed k-means clustering via sklearn.cluster.KMeans implemented in the python library sklearn. We chose *k* = 3, to be able to compare with the three TDA-based groups.

## Results

### Stratification of cohort 1 by TDA based on symptom levels at baseline

Cohort 1 was composed of 101 patients treated in an early intervention program (TIPP^2^). Patients were primarily males, with a mean age of 25.1 years and a mean illness duration of 1.9 years at baseline (Table 1). Most patients were prescribed antipsychotics (95%).

**Table 1:** Demographics.

	Cohort 1 (*n* = 101) mean [range]	Cohort 2 (*n* = 93) mean [range]	*p* value (test statistic)
% Male (male/female)	76.2% (77/24)	55.9% (52/41)	*p* = 0.005 (*χ*^2^ = 8.09)
Age at evaluation (years)	25.1 [17.1–38.2]	25.4 [18–35]	*p* = 0.75 (*t* = −0.32)
Illness duration (years)	1.9 [0–5]	2.0 [0–18]	*p* = 0.82 (*t* = −0.22)
Age at psychosis (years)	23.1 [13.6–38.0]	23.5 [14–35]	*p* = 0.68 (*t* = 0.42)

**Fig. 1:**
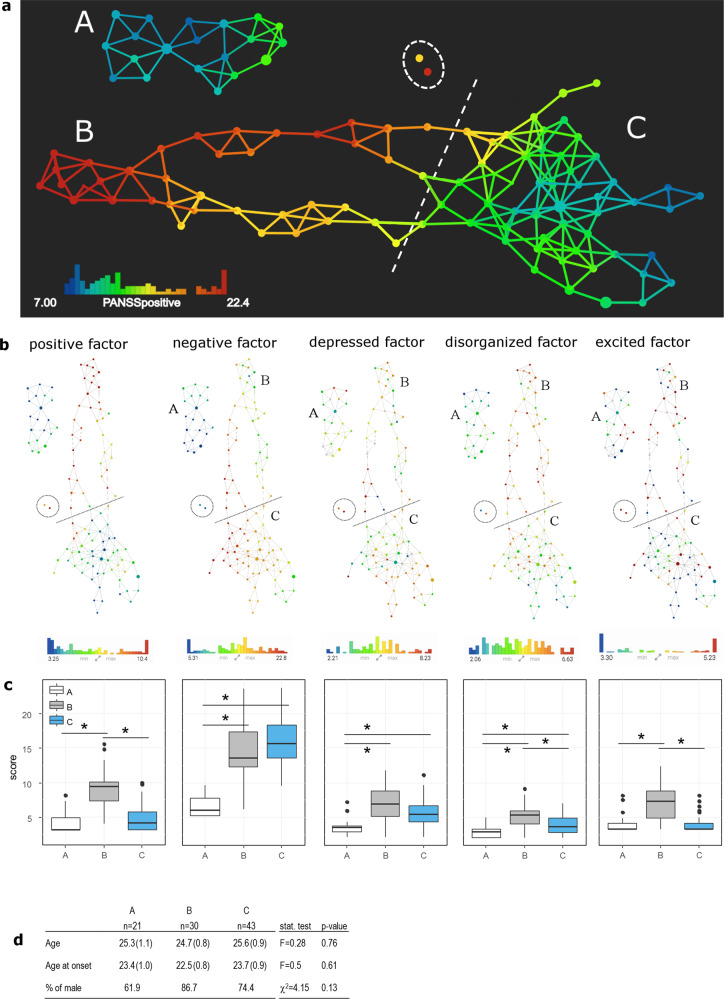
Identification of three clinical profiles at baseline. **a** Mapper graph based on the symptoms of early psychosis patients. Each node represents a group of patients. Each edge represents at least one common patient between the two connected nodes. Nodes are colored according to mean PANSS positive score. The dashed line represents the separation between groups B and C. The dashed circle highlights the two isolated nodes (*n* = 4 patients in total) who were not assigned to any group. **b** The Mapper graphs are colored according to the scores for the subscales of the Wallwork five-factor model. **c** Box plots illustrating the levels of symptoms at recruitment in groups A (white), B (gray), and C (blue) for the subscales of the Wallwork five-factor model. *p < 0.05 **d** Demographic characteristics of groups A, B, and C. Data are presented as mean (standard error).

We used the Mapper algorithm to visualize the data quantifying the severity of symptoms. We associated to each patient a vector of 30 entries recording the PANSS items’ scores. The Mapper algorithm was then used to cluster the vectors, forming the nodes of a graph. A vector can belong to more than one node; if two nodes share at least one vector, they are connected by an edge. By construction, vectors representing patients with similar symptom values at baseline belong to the same node or to a set of tightly connected nodes of the Mapper graph (Fig. 1a).

From the shape of the Mapper graph generated from the PANSS items, we identified three groups of patients: group A (*n* = 21), group B (*n* = 30), and group C (*n* = 43; Fig. 1a), with similar mean age, age at illness onset, and proportion of males/females (Fig. 1d). A small number of patients were not classified (*n* = 7, Supplementary Table 2). At recruitment, positive and excited symptoms were highest for group B (Fig. 1b, c); patients in group B had more elevated scores for many items of the positive and general PANSS than the rest of the cohort (e.g., P1, P3, G9; Supplementary Table 3). Negative and depressed symptoms were lowest in group A; low scores for negative and depressed symptoms characterized group A (e.g., N1, N2, G6). Higher scores on negative symptoms (e.g., N1, N2) distinguished group C from the rest of the cohort. Disorganized symptoms were high in group B, intermediate in group C, and low in group A.

We further explored blood metabolites, since the identification of biomarkers correlated with outcome might lead to the identification of target metabolic pathways.

### Biomarkers characterizing the clinical groups at baseline

Two groups of compounds were quantified at baseline: (1) redox-related metabolites and enzyme blood levels (GSH, GPx, GR, and Trx activities [34, 36]), and (2) 28 free amino acids and derivatives (Supplementary Table 4).

**Fig. 2:**
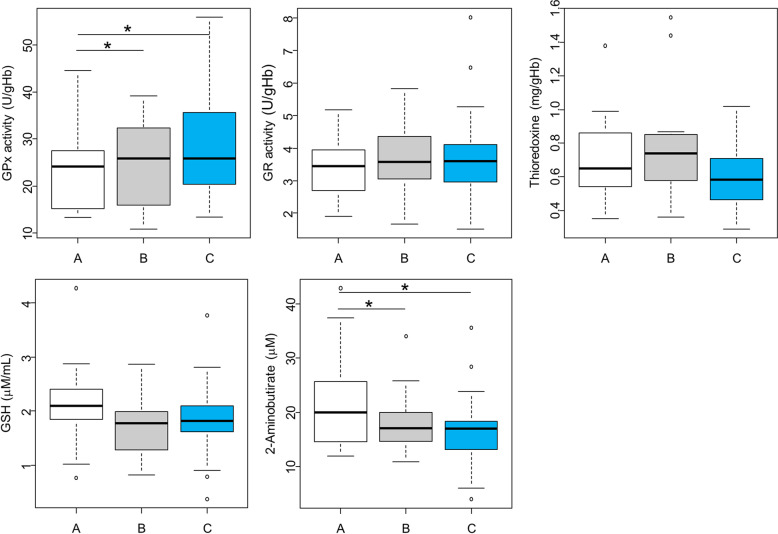
Metabolic characterization of the three clinical profiles at baseline. Boxplot illustrating the blood levels of three antioxidant enzymes (Glutathione peroxidase (GPx), Glutathione reductase (GR), Thioredoxin), of glutathione (GSH), and a modulator of GSH metabolism, 2-aminobutyrate. *p < 0.05 using a linear model adjusted for age and sex.

**Fig. 3:**
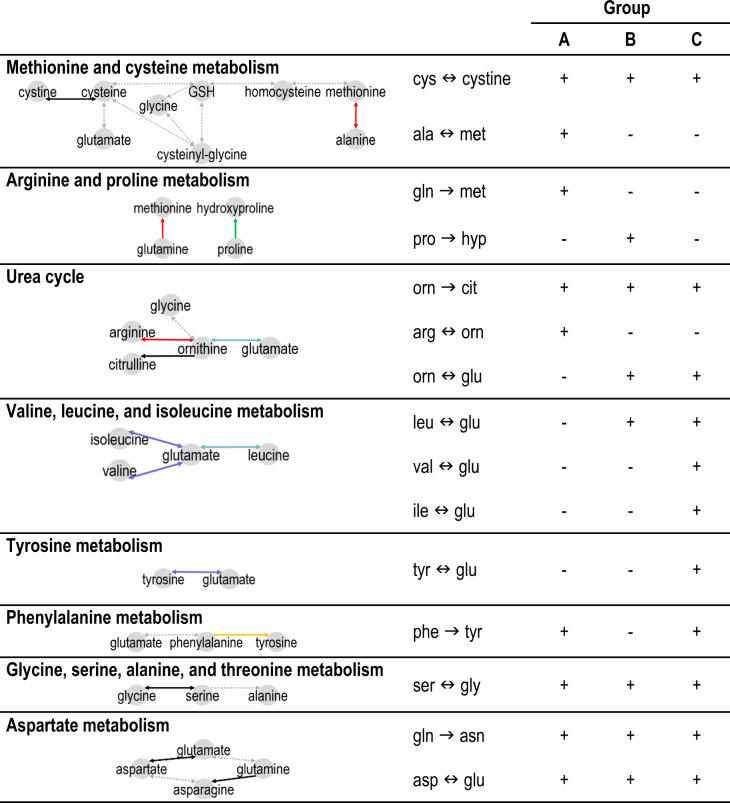
Metabolic pathways and the corresponding metabolites that are correlated for each clinical group (all correlations are positive). → or ↔: uni- or bi-directional metabolic reaction. Colored edges connect pairs of metabolites that are strongly correlated among patients (*p* < 0.05) in group A (red), group B (green), group C (blue), group A and group C (orange), group B and group C (light blue), and in the three groups (black). Note that the pattern of correlated amino acids differed between groups, and stronger correlations among a larger group of amino acids were detected in group A than in groups B and C (Supplementary Fig. 2). When we consider only the correlations that correspond to known biological pathways. (https://www.vmh.life/), the regulation of various systems appeared to be differentially impaired in the three groups. *hyp* hydroxy-proline, *cit* citrulline, *orn* ornithine.

GPx activity was lower in group A (23.3 ± 1.9 µmol/min/gHb) than in B and C, respectively (25.5 ± 1.9, *p* = 0.047 and 28.1 ± 1.6 µmol/min/gHb, *p* = 0.008; Fig. 2), while that of GR and Trx was similar in the three groups. Group A tended to have increased levels of the antioxidant GSH than group B (*p* = 0.062). Group A displayed higher 2-aminobutyrate levels (21.4 ± 2.1 µM) than B and C, respectively (17.9 ± 1.2 µM, *p* = 0.046 and 16.8 ± 1.0 µM, *p* = 0.009; Fig. 2). To further explore whether metabolic pathways related to these proteinogenic amino acids were differentially regulated in the three groups, we studied the correlations between metabolites that correspond to known biological pathways. In group A, the metabolism of nitric oxide and of methionine was affected (urea cycle and arginine, proline metabolism, Fig. 3 and Supplementary Fig. 2). Strikingly, many amino acids were correlated to glutamate levels in group C, possibly reflecting amino acid degradation, as the first step involves transamination to form glutamate [38].

### Predictive value of the grouping for the outcome at discharge

Clinical follow-up assessments included symptom levels, diagnosis, symptomatic remission [31], global and social functioning (GAF; SOFAS), and whether the patient was working or living independently at the end of 3 years of treatment [30].

**Fig. 4:**
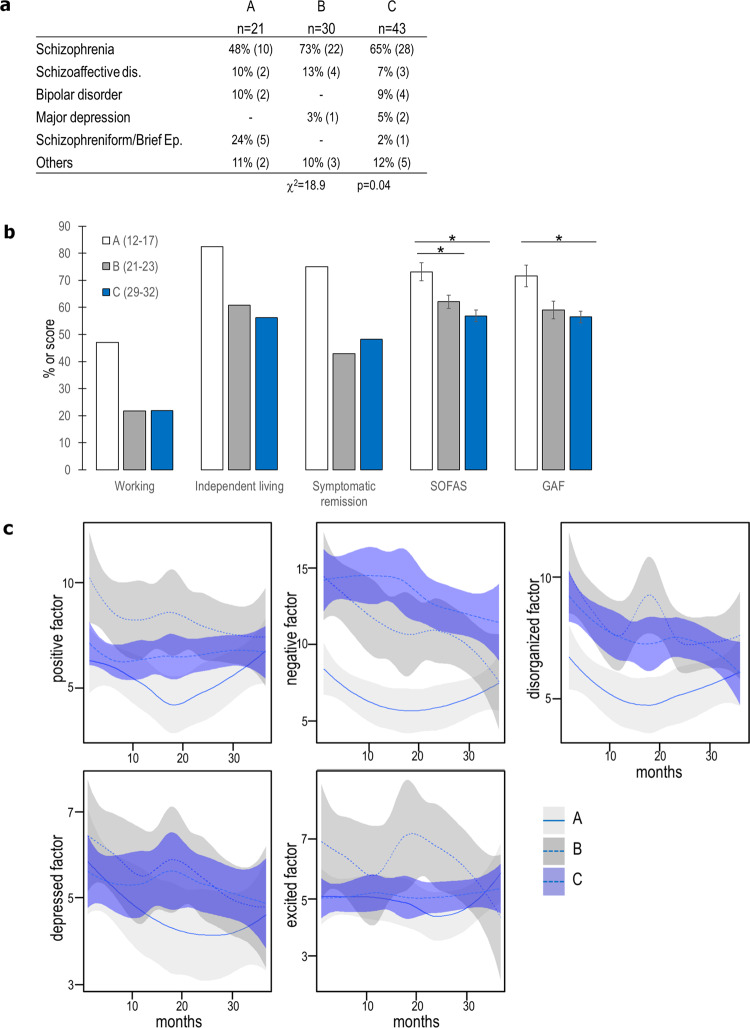
Outcomes associated with the three clinical profiles at discharge for cohort 1. **a** Table summarizing the retained diagnosis. The Chi-Square test for independence indicates a difference among the groups in the proportion of diagnosis. Brief Psychotic Ep.: brief psychotic episode. **b** Bar graph illustrating the percentages of patients from groups A, B, and C who are working, living independently, or in symptomatic remission, and the score for the SOFAS (Social and Functional Assessment scale) and GAF (Global Assessment of Functioning) scales. *p < 0.05. **c** Temporal evolutions of symptoms in cohort 1. Scores for the subscales of the Wallwork five-factor model in groups A (light gray), B (dark gray), and C (blue).Lines: Mean value, Shaded color: 95% confidence interval.

At follow-up, most symptoms had reached similarly low levels in all three groups (Fig. 4c); the exceptions were the negative symptoms, which remained higher in group C (12.6 ± 1.2) than in group A (7.4 ± 1.0; *p* = 0.005). Patients from group B were more frequently diagnosed with schizophrenia (Fig. 4a) and those from group A with schizophreniform disorder or brief psychosis episode. Patients from group A had better social functioning (SOFAS) than groups B and C, and better global functioning (GAF) than group C. (Fig. 4b). Varying the position of the boundary between groups B and C did not affect the profiles specific to each group at inclusion, and mostly preserved the differences of outcome (Supplementary Fig. 3).

We used machine learning with logistic regression to quantify the predictive power of this new stratification for good or poor psychosocial functioning at outcome (i.e., GAF > 65 or ≤65). Logistic regression was performed on either PANSS scores at baseline or membership in groups A, B, and C (Supplementary Table 1). The accuracy of logistic regression using the PANSS items was poor (precision: 0.20; recall: 0.05), but was 0.74 when using group membership instead (precision: 0.68; recall: 0.46; Supplementary Fig. 4c).

In summary, TDA of PANSS symptoms at baseline identified three clinically distinct groups of early psychosis patients with predictive value for functional outcome at 3 years.

### Validation study in cohort 2

A replication cohort of 93 TIPP patients was constituted (cohort 2), matched for age at illness onset, illness duration, and age at baseline; its proportion of females was higher (Table 1).

**Fig. 5:**
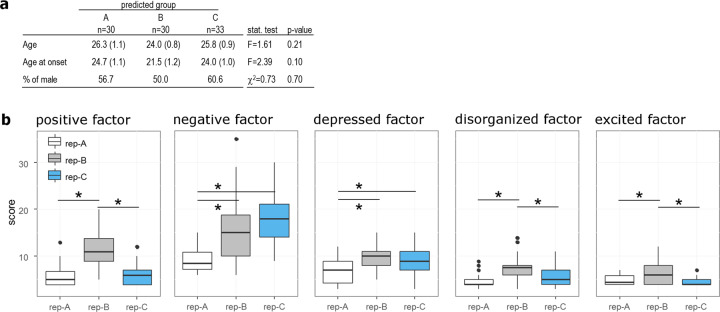
Replication of the three clinical profiles in cohort 2. **a** Table summarizing the demographic characteristics of the predicted groups in the validation cohort 2. Data are presented as mean (standard error). **b** Bar graph representing the levels of symptoms at recruitment in predicted groups A (white), B (gray), and C (blue) using Wallwork five-factors model. *p < 0.05.

Patients from the cohort 2 were assigned to replication groups based on the distance of their symptom vectors to the centroids of groups A, B, and C (Supplementary Fig. 5). For instance, a patient whose symptom vector was closer to the average symptoms in group A than to that of the other groups was assigned to replication-group A (rep-A). For the three replication groups from cohort 2, the number of patients, mean age, age at illness onset, and proportion of males/females were similar to those of cohort 1 (Fig. 5a). The baseline symptoms characterizing each group were reproduced in cohort 2 at the factor level (Fig. 5b) and at the item level (Supplementary Table 3).

### Outcome in the validation study

**Fig. 6:**
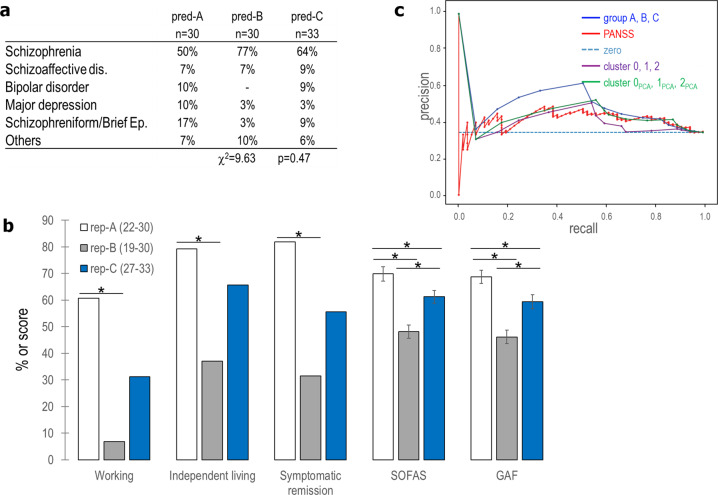
Replication of the outcomes associated with the three clinical profiles. **a** Table summarizing the retained diagnoses of patients in the validation cohort. The Chi-Square test for independence indicates a difference between groups in the proportion of diagnosis. Brief Ep.: brief psychotic episode. **b** Bar graph illustrating the percentage of patients in the validation cohort from predicted groups A, B, and C who are working, living independently, or in symptomatic remission, and the score for the SOFAS and GAF scales. *p < 0.05. **c** Precision-recall curve of Logistic Regression, with regularization strength 1, when using PANSS items (red), TDA-based group membership (blue), k-means clustering (purple), or k-means clustering on PCA (green). The dashed line represents the proportion of patients with good outcome in cohort 2, i.e., the precision of the model that always predicts good outcome.

The outcomes of patients in rep-A were better than those of rep-B for all the criteria assessed (Fig. 6b). Mean functioning scores (GAF, SOFAS) were different among the three groups: lowest in rep-B and highest in rep-A.

The group rep-A had the best outcome after 3 years of clinical follow-up, while rep-B had the worst outcome, as in cohort 1 (see Fig. 4b for comparison). There were two significant differences from cohort 1: (i) the retained diagnoses were similar among the predicted groups (Fig. 6a); (ii) the group rep-C had a better outcome than group C, displaying an intermediate profile, between rep-A and rep-B.

We trained logistic regression to predict good vs. poor functional outcome on cohort 1 and tested it on cohort 2. Using TDA-based groups as features, rather than PANSS items, we obtained better accuracy, precision, and recall scores (Supplementary Fig. 4a). The area under the precision-recall curve for PANSS items was 0.46, compared with 0.61 for the TDA-based groups (Fig. 6c). Overall, we validated with cohort 2 the clinical and demographic profiles of cohort 1. Logistic regression confirmed the robust predictive value of group A for long-term functional outcomes.

### Comparing topological clustering to a standard clustering method

To assess the originality of our clustering and confirm that we identify a robust structure in the data, we compared the TDA-based stratification of cohort 1 into groups A, B, and C, to the partition [0, 1, 2] or [0_PCA_, 1_PCA_, 2_PCA_] obtained by clustering the same data with the k-means algorithm (*K* = 3) or by clustering the two first principal component scores of the data with k-means (*K* = 3).

The k-means clusters [0, 1, 2] partly overlapped with the TDA-based groups (Supplementary Fig. 6). However, the predictive power of the partition [0, 1, 2] was lower than that of the TDA groups [A, B, C], according to all the metrics considered to evaluate the performance of logistic regression (Fig. 6c and Supplementary Fig. 4). The k-means clustering on PCA produces intermediate results between the k-means and the TDA-based groups. The clusters [0_PCA_, 1_PCA_, 2_PCA_] partly overlapped with the TDA-based groups (Supplementary Fig. 7). The predictive power of the partition [0_PCA_, 1_PCA_, 2_PCA_] was good when using cohort 1 as training set and cohort 2 as test set, but lower than that of the TDA groups [A, B, C] for the fivefold cross validation scheme (Fig. 6c and Supplementary Fig. 4).

## Discussion

Applying TDA to stratify a cohort of early psychosis patients (*n* = 101) according to their symptom levels, we identified three clinical profiles displaying distinct biological characteristics and different long-term functional outcome. Group A was characterized by an overall low level of symptoms and favorable functional outcome, while group B (with high positive and negative symptoms), and group C (with high negative symptoms) had poorer functional outcome. In a validation cohort (*n* = 93), we confirmed that predicted group membership is associated with distinct clinical profiles at baseline and at follow-up. TDA grouping also predicted good vs poor functional outcome with higher accuracy than the PANSS items or groups obtained by k-means clustering, confirming the clinical relevance of the TDA grouping.

Regarding redox markers, group A displayed a distinct metabolic profile, with the lowest GPx activity compared with groups B and C. As high blood GPx activities are associated with low brain GSH levels [37], this result suggests that better outcome within group A may be related to better redox regulation and poorer outcome in B and C to high oxidative status and/or an important redox dysregulation [18, 36]. Interestingly, the exploratory profiling of amino acids highlighted that 2-aminobutyrate levels were higher in group A than in B and C. 2-Aminobutyrate, proposed as marker for GSH dynamics [39–41], is a byproduct of cysteine, the limiting precursor of GSH, which modulates GSH homeostasis. These observations suggest a correlation between better regulation of antioxidant defense and better outcome (Supplementary Fig. 8). The correlations between urea cycle metabolites differed between group A versus B and C, suggesting a difference in reactions distributed between the mitochondrial matrix and the cytosol. The previously reported urea cycle dysregulation, as well as decreased arginine levels and nitric oxide imbalance in schizophrenia, may thus be more prominent in subgroups B and C [22, 24, 42].

Interpretation of the metabolic profile is hindered by our selection, which was oriented toward the assessment of redox balance. Inflammatory markers, lipid metabolism, NMDA receptor reactivity, and dopamine functions should also be examined, as should the potential effect of medication, since antipsychotics may alter amino acid metabolism [21]. Moreover, replication is needed to strengthen these conclusions, as blood was not available in our validation cohort.

The robustness of the TDA-identified groups was established by several tests: (i) the groups remained cohesive for different parameters and different implementations of the Mapper algorithm (Supplementary Fig. 1); (ii) profiles at inclusion and outcomes were stable when changing slightly the boundary between groups; (iii) most results were similar in both cohorts of early psychosis patients; and (iv) the k-means clustering stratification was similar to the TDA stratification, although k-means clusters overall had a lower predictive value for outcome. Compared with other stratification methods, an important advantage of Mapper is the graph structure visualization: connectivity variations when modifying the resolution and gain parameter were important for developing intuition about the relation between groups and their composition at different scales. Other group-assignment strategies that take into account the Mapper structure may improve group identification.

The finding that group A had better outcome was data driven and is therefore a robust finding. The low levels of symptoms in this group may indicate a good early response to treatment, associated with better functioning [11, 43, 44], and improved long-term outcome [45, 46]. The fact that patients with poor functional outcome split into two groups is also of importance, as different therapeutic strategies might be required, based on the clinical and metabolic profiles. Though group B was characterized by high positive symptoms at baseline, it did not include more treatment-resistant patients than those of group C, which present low positive symptoms at baseline (Supplementary Table 5). Furthermore, the observation that all three groups displayed similarly low levels of positive symptoms at the end of the clinical follow-up suggests that patients in group B are slow/poor responders to treatment and may need intensive case management or specific antipsychotic drug [8, 47]. Group B has neither patients with bipolar disorder nor with schizophreniform disorder and its less favorable outcome is in line with previous papers showing that schizophrenia has poorer outcome than affective psychoses or schizophreniform disorder [48, 49].

Negative symptoms are known to be associated with poor outcomes [50] and difficult to treat. Patients from groups B and C displayed high levels of negative symptoms at baseline, though these symptoms remain high only in group C. This may be linked to a different illness profile and neurobiological basis between groups B and C, which is actually in line with our results showing that they have distinct metabolic profiles. Group C may consist of patients with persistent trait-related negative symptoms, known indicators of poor psychosocial outcome [51–53]. The GPx activity was particularly high in group C, suggesting that they may benefit from antioxidant add-on therapy [35] such N-acetyl-cysteine, which improves negative symptoms [54, 55]. Despite the limitations of the tools used to assess negative symptoms our results suggest that on their basis it is possible to identify distinct subgroups of patents with distinct needs.

In summary, unsupervised data-driven topological analysis of symptoms produced meaningful stratification of early psychosis patients and detected patients at risk of poor functional and social outcomes. Although this study requires replication in patient populations recruited at different sites, this approach, combined with mechanism-based metabolic profiling, should pave the way for personalized functional-disability preventive strategies at early stages of the disease.

## Supplementary information

Supplemental material

Supplemental table 1
