# Supplementary material for: Topology predicts long-term functional outcome in early psychosis
Source: Mol Psychiatry. 2020 Jul 6;26(9):5335–46. doi: 10.1038/s41380-020-0826-1 (PMC8589664; doi:10.1038/s41380-020-0826-1)
Supplement: Supplementary file 1 — Supplemental material [file 41380_2020_826_MOESM1_ESM.pdf]

Supplementary Information

**Supplementary Tables 2 - 5**	**p.2 - 6**
**Supplementary Figures 1 - 8**	**p.7 - 17**
**Supplementary Methods**	**p.18 - 26**
**Supplementary References**	**p.27**

		Patient 4	Patient 10	Patient 78	Patient 164	Patient 179	Patient 181	Patient 182
Reason for not being classified		isolated node	isolated node	shared in B-C	shared in B-C	isolated node	shared in B-C	isolated node
Clinical profile at inclusion	Positive factor	7.7	9.1	7.4	8.9	4.9	6.7	16.3
	Negative factor	8.0	21.8	19.2	7.0	14.0	5.2	5.2
	Disorganized factor	2.9	4.5	6.2	2.1	4.5	2.1	8.3
	Excited factor	4.9	6.0	7.5	5.6	3.3	6.3	6.6
	Depressed factor	5.0	11.1	2.9	7.3	7.4	9.5	4.6
at outcome	Positive factor	10.9	5.8	3.3	7.5	5.1	6.5	#N/A
	Negative factor	6.9	23.6	22.6	7.9	6.2	6.2	#N/A
	Disorganized factor	6.2	2.5	3.7	5.0	5.8	2.5	#N/A
	Excited factor	3.3	3.3	5.0	10.9	5.1	8.9	#N/A
	Depressed factor	4.6	10.2	2.2	8.8	5.8	8.1	#N/A
	Working	0	0	0	0	0	0	#N/A
	Living indep.	1	1	0	1	0	1	#N/A
	Sympt. remission	0	0	0	1	1	1	#N/A
	SOFAS	30	35	45	59	60	70	#N/A
	GAF	20	35	45	59	60	70	#N/A

Supplementary Table 2. Clinical profile of the patients who were not classified into group A, B, or C. N/A: not available.

		Cohort 1	A	B	C	Cohort 2	rep-A	rep-B	rep-C
P1 [B]	Delusions	2.29 (0.15)	1.48 (0.2)	**3.77 (0.22)**	**1.65 (0.15)**	2.26 (0.14)	1.70 (0.15)	**3.47 (0.27)**	1.67 (0.11)
P2	Conceptual disorganization	1.68 (0.11)	1.29 (0.12)	2.27 (0.25)	1.47 (0.12)	1.86 (0.12)	1.53 (0.14)	2.63 (0.24)	1.45 (0.14)
P3 [B]	Hallucinations	1.98 (0.14)	1.52 (0.25)	**2.90 (0.27)**	1.56 (0.15)	2.02 (0.13)	1.37 (0.12)	**3.07 (0.27)**	1.67 (0.17)
P4	Excitement	1.63 (0.10)	1.48 (0.21)	**2.37 (0.21)**	1.19 (0.08)	1.34 (0.08)	1.57 (0.16)	1.47 (0.16)	1.03 (0.03)
P5	Grandiosity	1.50 (0.10)	1.05 (0.05)	2.03 (0.23)	1.35 (0.13)	1.34 (0.09)	1.10 (0.06)	1.83 (0.23)	1.12 (0.07)
P6 [B]	Suspiciousness/persecution	2.51 (0.15)	**1.52 (0.21)**	**3.87 (0.19)**	2.05 (0.17)	2.53 (0.15)	1.80 (0.19)	**3.77 (0.22)**	2.06 (0.20)
P7	Hostility	1.53 (0.10)	1.14 (0.08)	**2.13 (0.20)**	1.30 (0.13)	1.27 (0.06)	1.00 (0)	1.67 (0.15)	1.15 (0.08)
N1 [A, C]	Blunted affect	2.93 (0.15)	**1.52 (0.20)**	2.90 (0.26)	**3.63 (0.19)**	2.78 (0.16)	**1.67 (0.15)**	2.93 (0.31)	**3.67 (0.22)**
N2 [A, C]	Emotional withdrawal	2.70 (0.13)	**1.19 (0.11)**	2.87 (0.23)	**3.33 (0.15)**	2.30 (0.13)	**1.40 (0.12)**	2.43 (0.24)	**3.00 (0.18)**
N3	Poor rapport	2.14 (0.12)	**1.19 (0.11)**	2.33 (0.24)	2.47 (0.16)	1.82 (0.13)	1.47 (0.18)	2.07 (0.26)	1.91 (0.20)
N4 [A, C]	Social withdrawal	2.79 (0.15)	**1.19 (0.11)**	3.33 (0.27)	**3.19 (0.17)**	2.87 (0.15)	**1.73 (0.15)**	3.20 (0.29)	**3.61 (0.19)**
N5	Difficulty in abstract thinking	2.37 (0.13)	1.95 (0.25)	2.67 (0.24)	2.37 (0.21)	2.20 (0.12)	1.80 (0.14)	2.57 (0.26)	2.24 (0.17)
N6	Lack of spontaneity	2.30 (0.13)	**1.29 (0.14)**	2.30 (0.23)	2.79 (0.18)	2.17 (0.14)	1.47 (0.15)	2.23 (0.28)	2.76 (0.24)
N7	Stereotyped thinking	1.63 (0.09)	1.19 (0.13)	2.17 (0.18)	1.47 (0.11)	1.71 (0.10)	1.33 (0.12)	2.13 (0.22)	1.67 (0.14)
G1	Somatic concern	1.65 (0.12)	1.43 (0.21)	**2.47 (0.28)**	1.19 (0.09)	1.75 (0.11)	1.53 (0.15)	2.33 (0.26)	1.42 (0.12)
G2	Anxiety	2.97 (0.15)	2.10 (0.23)	**3.77 (0.25)**	2.84 (0.20)	3.18 (0.14)	2.60 (0.23)	3.77 (0.24)	3.18 (0.20)
G3	Guilt feelings	2.11 (0.13)	1.57 (0.22)	2.57 (0.24)	2.05 (0.19)	2.24 (0.12)	2.20 (0.19)	2.43 (0.22)	2.09 (0.20)
G4	Tension	2.29 (0.13)	1.62 (0.22)	**3.03 (0.24)**	2.09 (0.16)	2.48 (0.13)	2.13 (0.20)	3.03 (0.25)	2.30 (0.19)
G5	Mannerisms and posturing	1.85 (0.11)	1.19 (0.09)	2.30 (0.22)	1.86 (0.16)	1.43 (0.09)	1.20 (0.12)	1.77 (0.22)	1.33 (0.13)
G6 [A]	Depression	2.62 (0.15)	**1.24 (0.14)**	3.23 (0.26)	2.86 (0.21)	3.05 (0.16)	**1.97 (0.20)**	3.70 (0.31)	3.45 (0.23)
G7 [A]	Motor retardation	2.43 (0.11)	**1.29 (0.10)**	2.53 (0.17)	2.91 (0.15)	2.27 (0.13)	**1.50 (0.14)**	2.43 (0.24)	2.82 (0.20)
G8	Uncooperativeness	1.22 (0.07)	1.00 (0.00)	1.63 (0.18)	1.05 (0.03)	1.35 (0.07)	1.10 (0.06)	1.73 (0.19)	1.24 (0.09)
G9 [B]	Unusual thought content	1.72 (0.11)	1.19 (0.11)	**2.53 (0.23)**	1.42 (0.09)	2.19 (0.13)	1.70 (0.18)	**3.13 (0.22)**	1.79 (0.17)
G10	Disorientation	1.28 (0.07)	1.00 (0.00)	1.50 (0.17)	1.26 (0.09)	1.52 (0.09)	1.30 (0.10)	1.63 (0.18)	1.61 (0.17)
G11	Poor attention	2.09 (0.11)	1.38 (0.15)	**2.70 (0.19)**	2.00 (0.17)	1.77 (0.11)	1.43 (0.15)	2.37 (0.22)	1.55 (0.16)
G12	Lack of judgment and insight	2.39 (0.14)	2.14 (0.30)	3.00 (0.28)	2.09 (0.18)	2.97 (0.16)	2.17 (0.22)	**3.93 (0.30)**	2.82 (0.22)
G13	Disturbance of volition	2.21 (0.11)	**1.43 (0.16)**	2.63 (0.22)	2.30 (0.13)	1.75 (0.10)	1.47 (0.14)	2.03 (0.22)	1.76 (0.12)
G14	Poor impulse control	1.55 (0.09)	1.29 (0.12)	2.13 (0.20)	1.28 (0.09)	1.33 (0.08)	1.23 (0.10)	1.67 (0.18)	1.12 (0.08)
G15	Preoccupation	1.60 (0.10)	1.14 (0.10)	2.20 (0.22)	1.40 (0.12)	1.29 (0.08)	1.07 (0.05)	1.70 (0.20)	1.12 (0.07)
G16 [B]	Active social avoidance	2.33 (0.14)	**1.14 (0.10)**	**3.10 (0.23)**	2.37 (0.19)	2.46 (0.15)	1.57 (0.14)	**3.53 (0.30)**	2.30 (0.19)

Supplementary Table 3. Scores for each PANSS item in cohort 1 and 2 and the corresponding TDA-derived groups (data are presented as mean (standard error)). Superscript letters indicate in which group the corresponding PANSS items is specifically altered vs the rest of the cohort both in cohorts 1 and 2. Values in bold indicate differences between the median value of the group and the median value of the rest of the corresponding cohort for p-value<0.05 (Kolmogorov-Smirnov test with Bonferroni correction). Note that at recruitment, patients from group rep-A displayed fewer symptoms than patients from the other groups, while patients from rep-B were the most severely affected (Fig. 5b). Positive, disorganized, and excited symptom levels in rep-C were similar to those in rep-A; negative and depressed symptom levels were similar in rep-C and rep-B. At the item levels, most of the group characteristics from cohort 1 were reproduced in cohort 2. The coherence between the description of the groups by the single item model displayed in Table 1 and the five-factor model on the one hand, and the coherence between the description of the groups in cohort 1 and cohort 2 on the other hand, confirmed the stability of the clinical profiles of groups A, B, and C. Moreover, it indicates that each group's centroid is a good representative of the group's clinical profile, i.e., the symptom vectors in each group do not largely vary from the group's mean.

	A<br>n=18	B<br>n=21	C<br>n=33-34
Alanine	337.0 (25.34)	329.8 (15.12)	367.6 (12.85)
Arginine	75.5 (3.71)	83.9 (4.26)	74.4 (2.72)
Asparagine	43.1 (2.44)	44.6 (1.79)	41.8 (1.48)
Aspartate	3.4 (0.25)	3.4 (0.25)	3.7 (0.23)
Citrulline	25.5 (1.16)	29.1 (1.25)	28.0 (0.97)
Cysteine	253.3 (10.07)	253.0 (7.49)	244.1 (5.83)
Cysteinyl-glycine	36.3 (1.81)	38.5 (1.75)	40.9 (1.48)
Cystine	41.1 (3.27)	45.5 (1.77)	39.5 (2.29)
Glutamate	43.3 (4.8)	45.1 (4.45)	56.0 (5.17)
Glutamine	485.8 (15.79)	528.3 (10.95)	512.5 (13.59)
Glycine	203.7 (16.25)	215.4 (10.48)	201.4 (8.01)
Histidine	78.9 (2.62)	78.0 (2.1)	79.8 (1.67)
Homocysteine	9.9 (0.62)	11.6 (1.1)	9.7 (0.76)
Hydroxyproline	11.8 (1.3)	15.9 (1.36)	15.1 (1.25)
Isoleucine	65.9 (4.75)	61.5 (2.85)	69.6 (4.06)
Leucine	127.4 (8.51)	123.1 (4.85)	133.0 (6.4)
Lysine	165.1 (6.32)	159.6 (7.69)	157.6 (5.22)
Methionine	24.5 (1.59)	24.2 (1.22)	24.3 (0.99)
Ornithine	44.4 (3.6)	50.8 (3.01)	48.4 (2.24)
Phenylalanine	53.8 (1.95)	43.5 (4.25)	49.6 (2.67)
Proline	183.5 (15.12)	209.2 (14.04)	221.4 (11.22)
Serine	96.1 (5.48)	102.6 (3.36)	94.6 (3.21)
Taurine	75.7 (12.36)	75.2 (8.34)	62.3 (2.95)
Threonine	121.9 (7.64)	120.5 (5.73)	119.8 (5.14)
Tyrosine	60.5 (4.24)	60.8 (3.12)	64.6 (2.68)
Valine	224.1 (10.97)	217.2 (7.49)	245.2 (10.05)
2-Aminobutyrate	21.4 (2.07)	17.9 (1.18)	16.8 (0.98)
3-Methylhistidine	3.5 (0.33)	3.6 (0.38)	4.0 (0.26)

Supplementary Table 4. Metabolic profiling for amino acids and derivatives: Ala, Arg, Asn, Asp, Cit, Cys, Cysteinyl-glycine (Cys-gly) Cystine, Gln, Glu, Gly, Homocysteine (Hcy), His, hydroxy-Pro (Hyp), Ile, Leu, Lys, Met, Orn, Phe, Pro, Ser, Taurine, Thr, Tyr, Val, 3-methyl-His (3-mHis), 2-aminobutyrate (Abu). Values are presented as mean in micromoles per liter, with standard error in brackets.

	A (n=17)	B (n=22)	C (n=33)
**Ever having taken clozapine**			
Yes	0%	23%	15%
Considered	0%	0%	0%
No	100%	77%	85%
Unknown	0%	0%	0%
		χ^2=4.22	p=0.121
**Ever shown any response to a non-clozapine antipsychotic**			
Yes	94%	59%	85%
No	6%	36%	15%
Unknown	0%	5%	0%
		χ^2=9.17	p=0.057

Supplementary Table 5 Evaluation of the proportion of treatment-resistant patients in groups A, B, C of cohort 1. Treatment resistance was defined based on clozapine treatment (‘Ever having taken clozapine’) and response to non-clozapine anti-psychotics (‘Ever shown any response to a non-clozapine antipsychotic’).

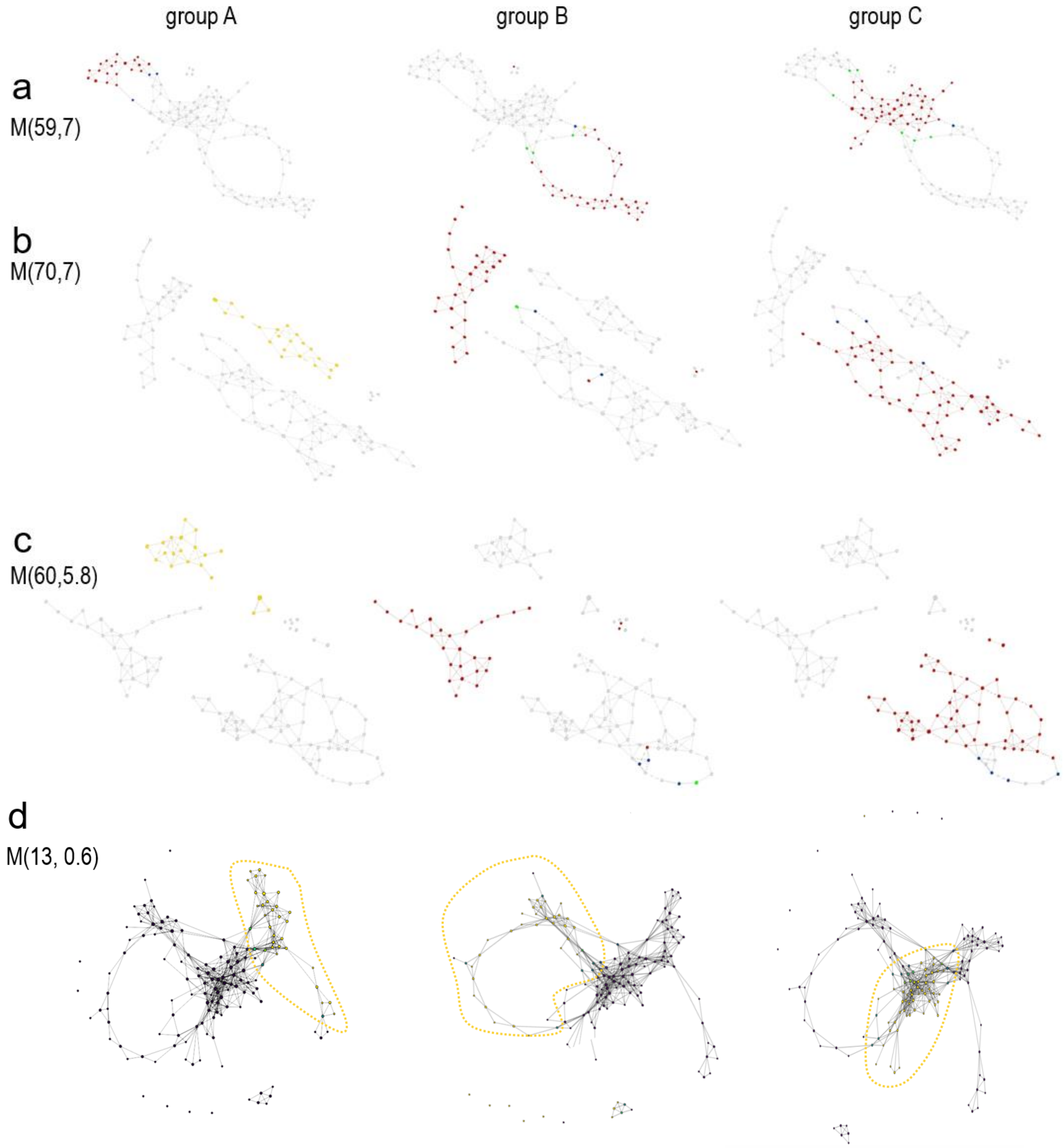

Supplementary Fig. 1 Effect of different resolution and gain on Mapper graph structure and of different implementations. The Mapper algorithm (see Supplementary Methods, section Mapper Algorithm), is a sequence of three steps: i) bin data into overlapping intervals, ii) cluster within each interval, ii) create a graph where vertices are clusters and an edge connects clusters that have points in common. The number of intervals and their overlap are the so-called *resolution* and *gain* parameters and are indicated in brackets for the settings

presented in panels **a-d**. In the main Figure 1, Mapper settings are M(70, 6) Lower resolution and higher gain lead to more connected graphs (e.g., compare panels **a** and **b** or **a** and **c**). **a-c.** Maps were generated used Ayasdi's implementation of Mapper and different resolution and gain as indicated. **d.** Map generated using the open-source kepler mapper (https://kepler-mapper.scikit-tda.org/) with the following parameters: the first two principal components of the data as filter function, hierarchical clustering based on Pearson correlation distance, complete linkage, number of clusters for each interval is two resolution (n_cubes) 13, gain (perc_overlap) 0.6.

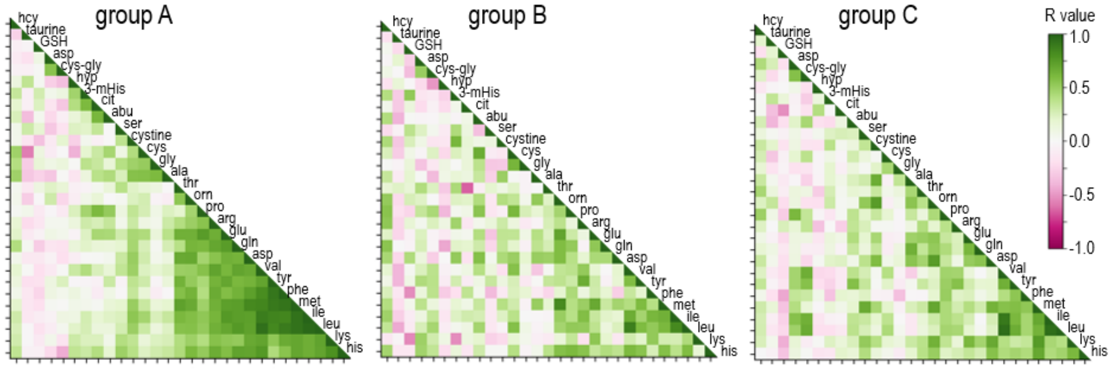

Supplementary Fig. 2 Correlation matrices representing Pearson correlation coefficients between blood levels of metabolites, measured in patients from groups A, B, and C. The stronger and larger correlations between amino acids in group A suggest that a better general metabolic homeostasis regulation prevails, compared to groups B and C.

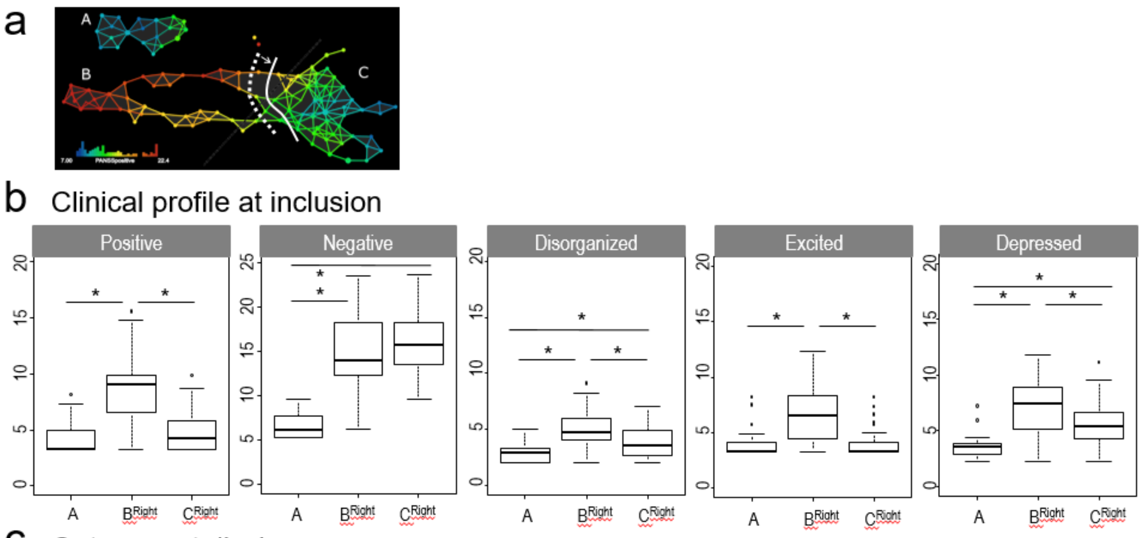

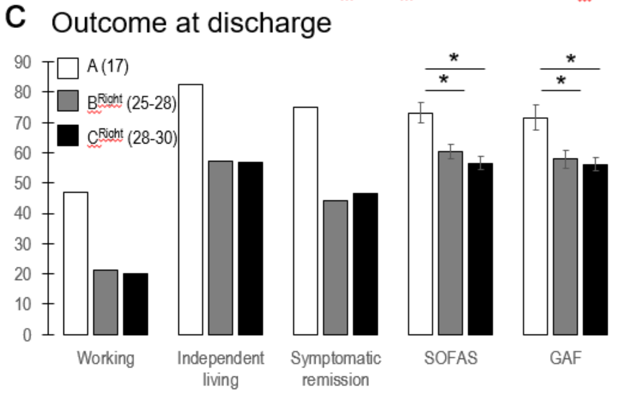

d

	A	BRight	CRight
	n=21	n=36	n=40
Schizophrenia	48% (10)	75% (27)	65% (26)
Schizoaffectif disorder	10% (2)	11% (4)	7.5% (3)
Bipolar disorder	10% (2)	3% (1)	7.5% (3)
Major depression	-	3% (1)	5% (2)
Schizophreniform/Brief Ep.	24% (5)	-	2.5% (1)
Others	10% (2)	8% (3)	12.5% (5)
		χ^2=22.5	p=0.069

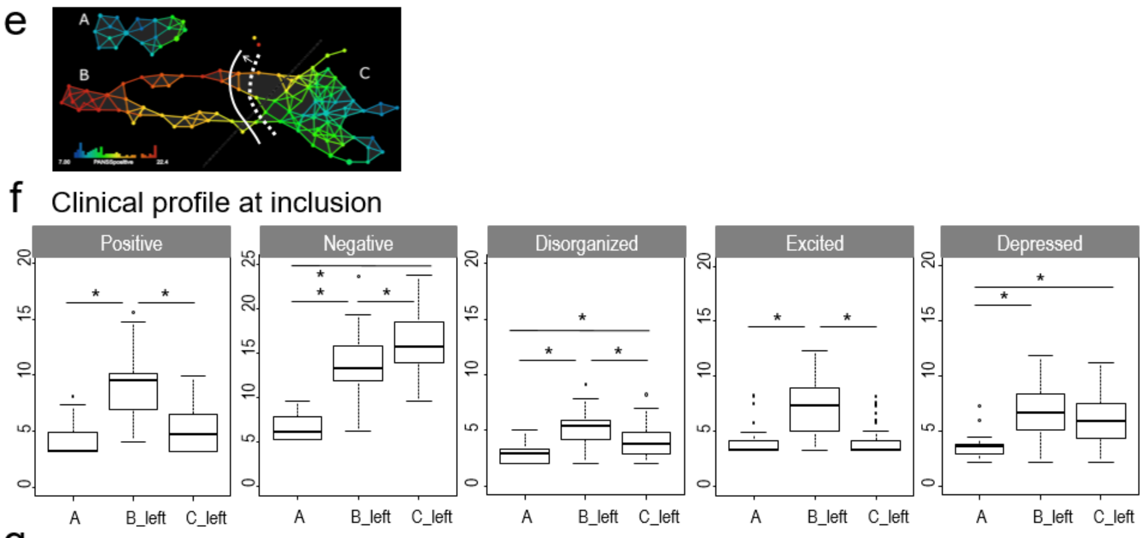

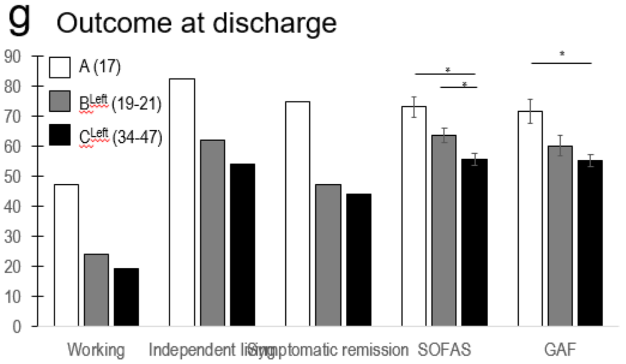

h

	A	BLeft	CLeft
	n=21	n=27	n=49
Schizophrenia	48% (10)	74% (20)	67% (33)
Schizoaffectif disorder	10% (2)	15% (4)	6% (3)
Bipolar disorder	10% (2)	-	8% (4)
Major depression	-	4% (1)	4% (2)
Schizophreniform/Brief Ep.	24% (5)	-	2% (1)
Others	10% (2)	7% (2)	12.5% (6)
		χ^2=24.9	p=0.035

Supplementary Fig. 3 Effect of moving the boundary between groups B and C on the clinical presentations and 3-year outcomes. **a, e** The boundary was moved towards the right or towards the left respectively, thus increasing the size of group B, by defining B^{Right} or decreasing the size of B by defining B^{Left} (and conversely decreasing the size of group C with C^{Right} or increasing the size of C with C^{Left}). Group A remains unchanged in both cases. **b, f** Box plots illustrating the levels of symptoms at recruitment in group A (white), B^{Right} or B^{Left} (grey), and C^{Right} or C^{Left} (black) using Wallwork five-factors model of the PANSS items. *: $p<0.05$. **c, g** Bar graph illustrating the percentage of patients who are working, living independently, in symptomatic remission, and the score for the SOFAS and GAF evaluations. *: $p<0.05$. **d,h** Table summarizing the retained diagnosis. The Chi-Square test for independence indicates a difference among the groups in the proportion of diagnosis. Brief Psychotic Ep.: brief psychotic episode.

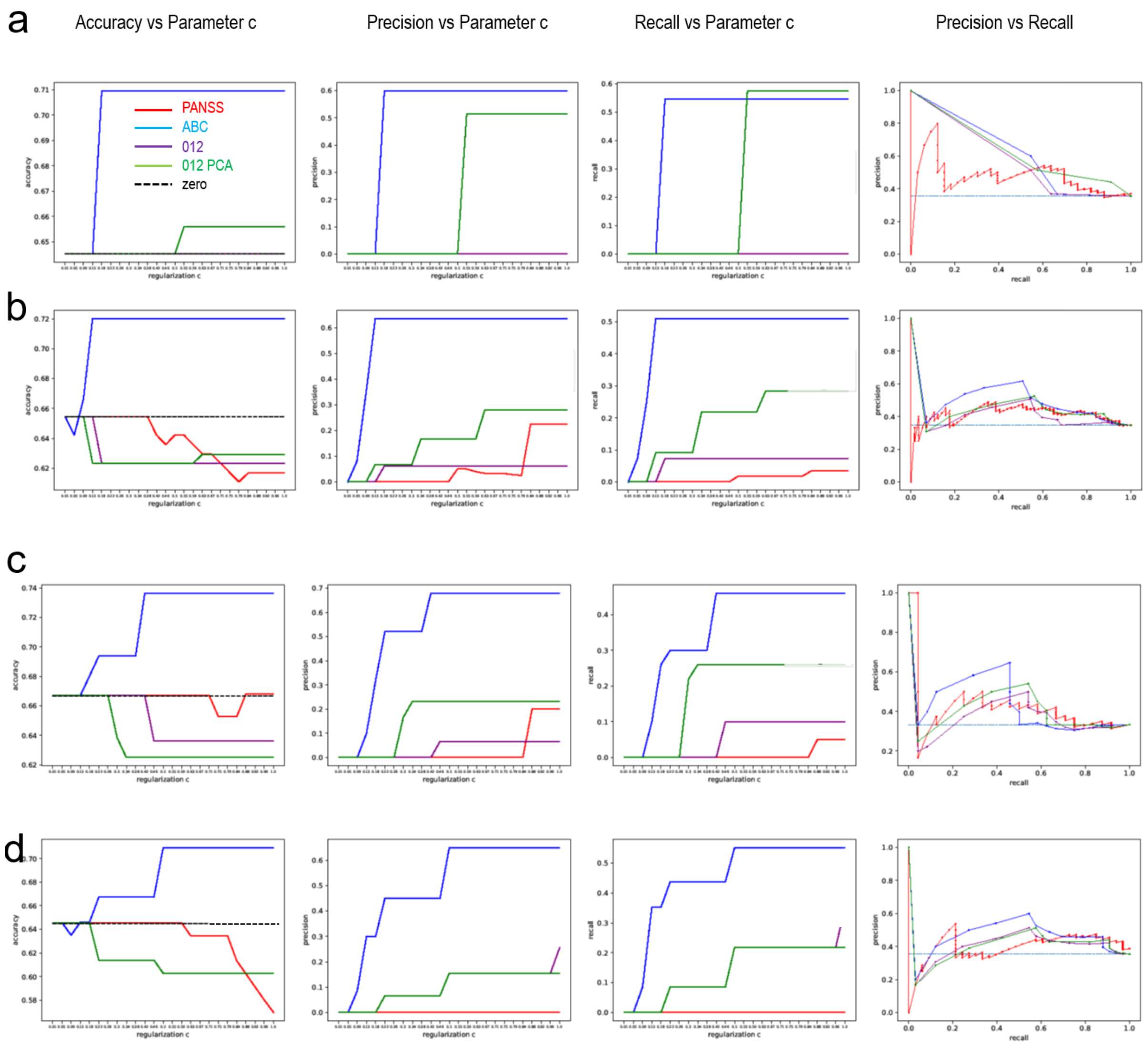

Supplementary Fig. 4 Accuracy, precision, recall, and precision-recall curves of logistic regression, as a function of the variation of the parameter c, which measures the inverse of the regularization strength. The binary prediction tasks consists in predicting good functional outcome (GAF >65) or poor outcome (GAF <=65), when using as a set of features PANSS items (red), TDA-based groups (blue), k-means groups (purple), k-means performed on PCA coordinates of the data (green). The black dashed line is the accuracy of always predicting poor outcome. The light blue dashed line is the proportion of patients with good outcome on the test set. The threshold parameter used to decide between good and poor outcome, given the probability of good outcome, is fixed to 0.5.**a** Training set of the model is cohort 1 and

test set is cohort 2. **b** 5-fold cross validation on the union of cohort 1 and cohort 2. **c** 5-fold cross validation on cohort 1. **d** 5-fold cross validation on cohort 2.

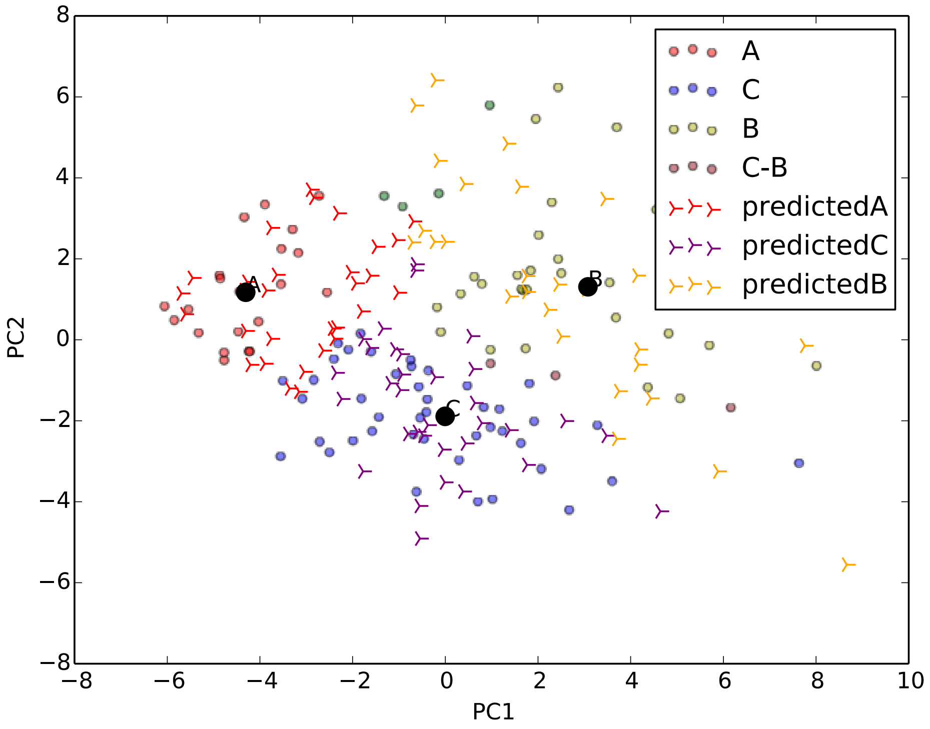

Supplementary Fig. 5 Cohesiveness of groups in cohort 1 and 2. Scatter plot of the first two principal component scores for the symptom vectors within group A (red circle), group B (yellow circle), group C (purple circle) and within predicted group A (red arrow), predicted group B (yellow arrow) and predicted group C (purple arrow). The first two principal component scores for the centroids of groups A, B and C are marked as black circles. In the coordinate systems of the first two principal components, group A and predicted group A are cohesive around the centroid of group A; similar behavior is observed for B and C.

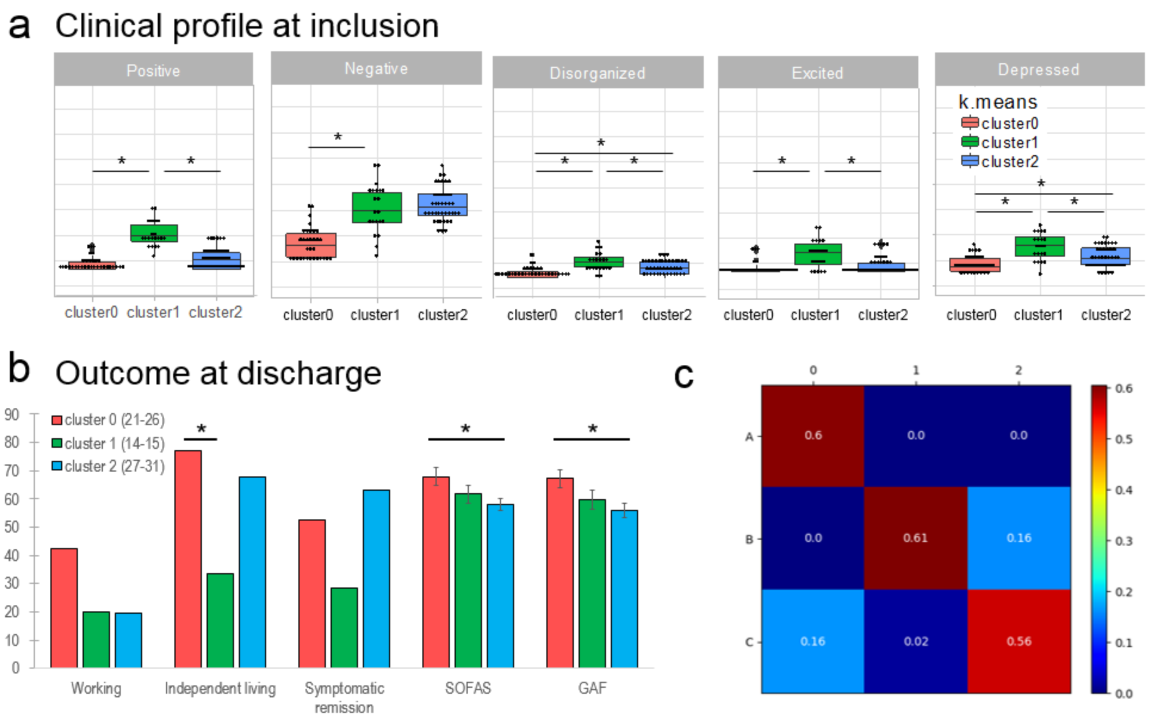

Supplementary Fig. 6 k-means clustering (compare with Fig. 1 & 2) **a** Box plots illustrating the levels of symptoms at recruitment in clusters 0 (red), 1 (green), and 2 (blue) using Wallwork five-factors model of the PANSS items. *: p<0.05. **b** Bar graph illustrating the percentage of patients who are working, living independently, in symptomatic remission, and the score for the SOFAS and GAF evaluations. *: p<0.05. **c** Similarity between groups A, B, C and 0,1,2. Matrix representing the Jaccard index between blocks in the partition A, B, C and the partition 0,1, 2 respectively. Group A was entirely contained in group 0, which also contained some elements from group C. Group 1 mainly contained elements from group B and a small percentage of elements from group C, while Group 2 mainly contained elements from group C and a small percentage of elements from group B.

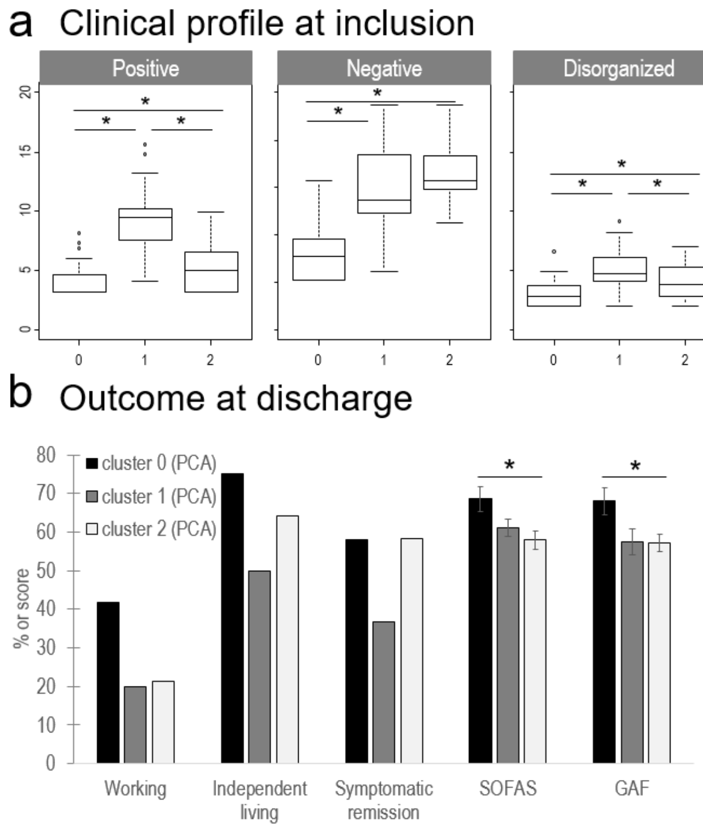

Supplementary Fig. 7 Description of the groups obtained by k-means clustering on PCA. **a** Box plots illustrating the levels of symptoms at recruitment in cluster 0 (white), 1 (grey), and 2 (black) using Wallwork five-factors model of the PANSS items. *: $p<0.05$. **b** Bar graph illustrating the percentage of patients who are working, living independently, in symptomatic remission, and the score for the SOFAS and GAF evaluations. *: $p<0.05$.

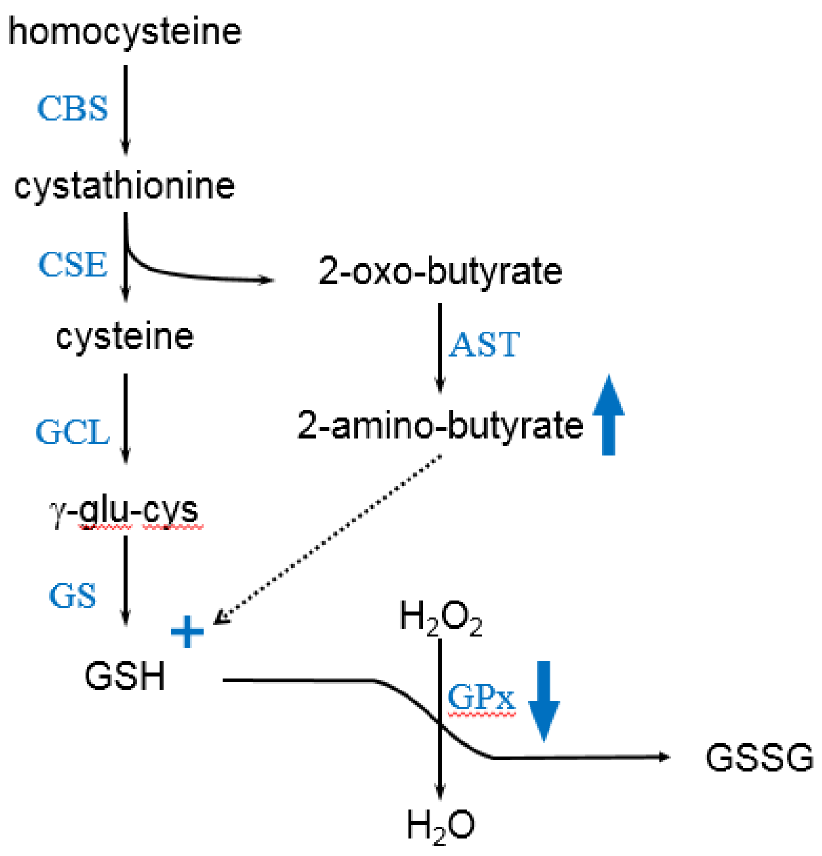

Supplementary Fig. 8 Regulation of GSH metabolism by 2-aminobutirate and changes in patients from group A. An upwards arrow indicates an increase in 2-amino-butyrate in group A compared to the other groups; a downwards arrow indicates a decrease in GPx activity in group A compared to the other groups. CBS: Cystathionine beta-synthase; CSE: cystathionine gamma-lyase; GCL: Glutamate cysteine ligase ; GS: Glutathione synthase; AST: aspartate aminotransferase; GPx: Glutathione peroxidase.

Supplementary Methods

Participants. All subjects provided a fully informed written consent according to the ethical guidelines of Lausanne University. All procedures were in accordance with the ethical standards of the Helsinki Declaration as revised in 1983 and approved by the ethical committee of Lausanne University Hospital on human experimentation.

The inclusion criteria for Treatment and early Intervention in Psychosis Program (TIPP[1]) are (i) to have a psychotic disorder, defined by reaching “psychosis threshold” subscale on the Comprehensive Assessment of At Risk Mental States scale (CAARMS[2]), (ii) for less than five years' duration. Neurological disorders and severe head trauma were exclusion criteria. Follow-up assessments exploring various aspects of treatment were conducted at baseline and after 2, 6, 12, 18, 24, 30 and 36 months in treatment. Diagnosis was based on the Diagnostic and Statistical Manual of Mental Disorders-Fourth Edition (DSM-IV) criteria[3]. Diagnosis was the result of an expert consensus by a senior psychiatrist and psychologist, who reviewed the patient's entire file over the treatment period (i.e., after 18 months and after 36 months)[4]. Cohort 1 comprises patients from the TIPP program (at baseline, the mean duration of treatment in TIPP was 10 months) who were recruited between December 2004 and February 2015 for a biomarker study. Cohort 2 comprises patients from the TIPP program (at baseline, the mean duration of treatment in TIPP was 7 months), who were recruited between October 2004 to February 2014 and who were not included in the biomarker study. Previous analyses revealed that patients included in the biomarker study were not less ill than those who refused (e.g., no differences between two with respect to symptoms and GAF scores at baseline), and therefore the patients in cohort 1 were globally representative of patients in the entire cohort[5].

Blood drawn for the assessment of metabolic markers. Blood was collected on EDTA-coated tubes and centrifuged at 3000g, 5 min, 4°C.The resulting plasma supernatant was aliquoted and stored at -80°C. The blood cell pellet was washed 2 times with 0.9% NaCl and

stored at -80°C. All manipulations were performed rapidly on ice to avoid artefactual oxidation of thiol compounds.

Mapper algorithm. We used the Mapper algorithm, in the Ayasdi implementation (www.ayasdi.com), to visualize and study the scores obtained by patients on the PANSS items. Mapper is a topological method, widely used for exploratory data analysis. This unsupervised algorithm performs multivariate pattern analysis of data as follows[6].

- Choose a notion of distance between data points and a function on the data with values in R^n (i.e., a *filter function)*.
- Cover the range of the filter function with overlapping open sets. The number of intervals and their overlap are the so-called *resolution* and *gain* parameters.
- Perform a clustering algorithm locally on each set of data points whose filter function values lie in an element of the cover. Each cluster will be represented by a node in the output graph.
- Connect two nodes in the output graph by an edge if they share at least one data point.

In our analysis, each data point is a vector reporting the severity of a patient's symptoms, according to the 30 PANSS items, at baseline (e.g., the vector for patient i is, P_i={$P1_i$, $P2_i$, … $G16_i$}, which corresponds to the coordinates of this patients in a 30-dimensional space). The distance we used between the data points is the normalized Pearson correlation distance, implemented in the Ayasdi software as one minus the Pearson correlation coefficient computed on the normalized data (i.e., each normalized data vector has mean 0 and standard deviation 1). Our filter function has values in R^2 and associates to each data point the coefficients in the coordinate system given by the first two principal components. Therefore, data points belonging to the same node or to tightly connected nodes are highly correlated according to Pearson correlation coefficient and their first two principal component in Principal Component Analysis are close in Euclidean distance. These choices of metric

and filter function are standard and allow to ‘spread out’ the data and highlight local structure. The same type of filter function was also used for instance in Bruno et al.[7]. We chose a resolution of 60 and therefore considered a cover of the range of the filter function given by 60 open squares. The gain parameter **g** in the Ayasdi implementation is a number between 1 and 10 that represents the percent of overlap **o** between opens of the cover, using the formula **o = 1-(1/g)**. Our choice of the gain value is 7.

Furthermore, we chose the equalized setting, so that each element of the open cover contains the same number of data points, and the gain measures the percentage of data points in the overlap between open sets of the cover. Since we are considering a high value of the resolution and gain parameters, the Mapper output graph has a large number of nodes and edges. Because a patient’s symptom vector will usually belong to many nodes, we did not focus on single nodes but rather on regions of connected nodes in the graph.

TDA stratification of cohort 1. From the shape of the Mapper graph, we identified three groups of patients we called A, B, and C. Each group of patients was selected by first distinguishing a group of nodes in the Mapper graph that exhibit remarkable connectivity and then listing the patients contained in those nodes. The nodes comprising group A were disconnected from the rest of the graph and therefore naturally form a distinctive sub-group. The nodes comprising groups B and C were chosen instead for the different types of connectivity they present. While group B is loosely connected, group C is more cohesive and tightly connected. Small variations of the resolution and gain parameter did not change the connectivity properties defining our groups (see Supplementary Fig. 1). Three nodes containing a total of four patients were isolated from the rest of the graph and thus were not attributed to any of the groups and excluded from our analysis. Although groups B and C do not have nodes in common, three vectors were contained in nodes of both groups; the corresponding patients were not assigned to either group and excluded from our analysis.

Stability of the groups based on the Mapper graph

We will now give an intuitive idea of how the Mapper graph changes when the resolution and gain parameters vary (Supplementary Fig. 1). The resolution **r** is the number of intervals into which we divide the range of the filter function and takes values between 1 and the number of data points. The gain **g** is a number between 1 and 10 that measures the proportion of data points in the intersection of two intervals. The remaining parameters that determine this construction are considered to be fixed.

We denote the Mapper graph with resolution r and gain g by M(r,g). In showing that the clusters A, B, and C are clearly visible in the Mapper graph for choices of the resolution and gain that produce interesting connectivity, we exclude extreme cases where there are many isolated nodes, many small connected components, or, at the opposite extreme, almost all the nodes are connected and contain the same set of patients. In this study we considered the graph M(60,7). These parameters allow us to define the three groups A, B, and C and in particular to identify A as a connected component in the Mapper graph, which in turn has a strong impact on the predictive power of our clustering.

If we slightly decrease the resolution and keep the gain constant, the graph M(59,7) has only one connected component. Visualizing our groups in this graph, we observe that A is adjacent to C, which is in turn adjacent to B (Supplementary Fig. 1a). This observation is coherent with the fact that the k-means algorithm clusters subgroups of C together with A. A single connected component with a similar structure is still visible when further reducing the resolution parameter. For small values of the resolution (as for example $r<5$), the Mapper graph has a trivial structure.

Let us now consider $r > 60$ and therefore obtain a graph with more nodes, while keeping the gain constant. The graph M(70,7) has three connected components, which almost perfectly correspond to A, B, and C (Supplementary Fig. 1b).

A different way to disconnect our original Mapper graph M(60,7) is to decrease the gain parameter. Similarly to M(60,7), the graph M(60,6) has two connected components, but the cycle present in group B becomes a flare (a branch), which correlates well with the severity of positive symptoms, with the patients with the most severe symptoms being the furthest from group C. The flare structure does not clearly correlate with values of GAF at outcome, however. For M(60,5.8) the graph already has three connected components, corresponding well to the partition [A,B,C] (Supplementary Fig. 1c).

The graph M(70,5) is already too fragmented to show cluster structure. It is worth noticing that the largest connected component in M(70,5) contains 32 patients who are all from group C. For r=70 and g<5, we also expect the graph to have too many components to present interesting connectivity structure.

Although similar in their composition, the three connected components obtained in M(60,5.8) and in M(70,7) do not contain exactly the same sets of patients. This is also true for other settings for which the Mapper graph has mainly three connected components, such as M(68,7) and M(69,7). The subdivision into two connected components and the greater cohesiveness of the region corresponding to group C compared to group B (which is represented either by a cycle or a flare) are instead more stable properties. For this reason we chose our partition [A,B,C] on a Mapper graph with only two connected components and separated B from C. An alternative strategy would have been to look for consensus in clustering among all the graphs with three connected components or even among all the graphs M(r,g)[8].

Validation of the stratification in cohort 2. We validated the predictive power of the clinical profiles identified in cohort 1 with an independent cohort of 93 individuals we called cohort 2. Given a partition P of cohort 1, based on the PANSS scores of patients, we defined a partition of cohort 2 as follows. First we computed the centroid of the symptom vectors of each group in P by considering the average value, within the group, of each coordinate in the

thirty-dimensional space of PANSS scores (e.g., coordinates of the centroid for group A are: $Centr_{A=}$($P1_A$, $P2_A$, … $G16_A$), where '$P1_{A'}$ is the mean of P1 items of the PANSS for patients in group A). For each patient in cohort 2, we then computed the Euclidean distance between his symptom vector and the centroids of the groups in P (e.g., distance of a new patient from the centroid of group A is: $Dist_{new,A}=\sqrt{(\mathrm{P1}_{new}-\mathrm{P1}_A)^2+(\mathrm{P2}_{new}-\mathrm{P2}_A)^2+\cdots+(\mathrm{G16}_{new}-\mathrm{G16}_A)^2}$). Each patient was assigned to the group with the closest centroid. This assignment determined a partition of cohort 2 into subgroups, which we called the replicated groups. In this study we determined replicated groups for the partitions [A,B,C] and [0,1,2] of cohort 1.

Prediction of good *vs* poor functional outcome. We used logistic regression to predict good *vs* poor functional outcome (GAF at outcome >65 *vs* GAF at outcome <=65), by using as a set of features either the PANSS items or group membership in either [A,B,C] or [0,1,2] . For patients in cohort 2, group membership means membership in the replicated groups (see methods '*Validation of the stratification in cohort 2*'). Membership in a given group is encoded with a binary variable: value 1 for individuals in that group and 0 for individuals who do not belong to that group. Note that only 72 individuals of the 101 in cohort 1 had information on GAF at outcome. To avoid imputing missing values for 28.7% of the data, we restricted to the subset with no missing values. On the other hand, all 93 patients in cohort 2 were assessed for GAF at outcome.

The proportion of patients with poor outcome in cohort 1 was 0.67. The proportion of patients with poor outcome in groups A, B, and C were 0.35, 0.74 and 0.78, respectively. The proportion of patients with poor outcome in groups 0, 1, and 2 were 0.5, 0.73 and 0.77, respectively. In the replicated groups A, B and C, the proportions of poor outcome were 0.4, 0.87 and 0.67, respectively. In replicated groups 0,1, and 2, the proportions of patients with poor outcome were 0.49, 0.86, and 0.67, respectively.

We applied three different validation schemes for our prediction model.

- 5-fold cross validation on cohort 1
- Training on cohort 1 and validation on cohort 2
- 5-fold cross validation on the combined data of cohort 1 and cohort 2

The performance of logistic regression was evaluated by three metrics: accuracy precision, and recall, as implemented in the sklearn library. We denote by t_p the number of true positives, f_p the number of false positives, t_n the number of true negatives, f_n the number of false negatives. Accuracy is defined to be $t_p+t_n/(t_p+f_p+t_n+f_m)$, precision is the ratio $t_p/(t_p+f_p)$ and recall is $t_p/(t_p+f_n)$. We recorded the values of these metrics as a function of the parameter c, which is the inverse of the regularization strength in the model. Smaller values of this parameter correspond to stronger regularization and therefore penalize large weight coefficients. We also considered the precision-recall curves, which are defined by interpolating between points in the plane the coordinates of which are the recall and precision of the model as a function of the threshold parameter. The threshold parameter determines the value of the predicted binary variable (good or poor functional outcome), given the predicted probability of good outcome. We used the function precision_recall_curve in sklearn to compute the precision-recall curves and the function sklearn.metrics.auc to compute the area under the curve.

Construction of the Metabolic map and selection of robust correlations. We took into account the biological interplay among 29 metabolites (i.e. 28 measured metabolites and GSH) by placing them in a metabolic map as nodes. There is a directed edge from a source node to a target node if the source node is among the substrates of a biological reaction and the target node is among its products. The list of chemical reactions in human metabolism was extracted from the virtual metabolic human database, which contains 13544 reactions (https://vmh.uni.lu/). Starting from this data, our metabolic map was constructed by following these steps.

-We built an edge list, where source and target nodes are among the 29 measured metabolites, and they are respectively a reactant and product in our reactions list. The length of this edge list was 2387, and the number of unique reactions involved in this edge list was 700.

- We excluded edges that were associated to ‘transport’ and ‘exchange’ reactions as they do not involve chemical reactions. The number of remaining edges was then 86. The number of involved reactions was 52.

- We selected edges corresponding to reactions with a confidence score of at least 2 (e.g., direct evidence from genome annotations, gene function, indirect evidence from physiological data, or direct evidence from biochemical study[9]). The final list includes 69 edges corresponding to 40 reactions.

We then focused on what we called strong biological correlations. To this end, for each metabolite we considered the vector of its expression levels in a group of patients. Two metabolites are considered strongly correlated in a group of patients if the corresponding Pearson correlation between the associated vectors is significantly different from zero (p-value < 0.05).

Among the 29 measured metabolites in cohort 1, we assessed which pairs were strongly correlated in groups A, B, or C. Presence of strong biological correlations in one or more groups corresponds to colored edges in our metabolic map, whose source and target are strongly correlated metabolites in that or those groups.

k-means clustering and Jaccard index.

The correspondence between the partitions [A, B,C] and [0,1,2] was assessed by comparing each block in the first partition to each block in the second partition through the Jaccard index. Given two sets X and Y, the *Jaccard index:*

$Jac(X,Y) := |X \cap Y| / |X \cup Y|$, the cardinality of the intersection of X and Y divided by the cardinality of their union.

Supplementary references

1. Baumann PS, Crespi S, Marion-Veyron R, Solida A, Thonney J, Favrod J *et al.* Treatment and early intervention in psychosis program (TIPP-Lausanne): implementation of an early intervention programme for psychosis in Switzerland. *Early Interv Psychiatry* 2013; **7**(3)**:** 322-328.

2. Yung AR, Yuen HP, McGorry PD, Phillips LJ, Kelly D, Dell'Olio M *et al.* Mapping the onset of psychosis: the Comprehensive Assessment of At-Risk Mental States. *The Australian and New Zealand journal of psychiatry* 2005; **39**(11-12)**:** 964-971.

3. American Psychiatric Association. Diagnostic and statistical manual of mental disorders: DSM-IV. *Washington (DC): American Psychiatric Association* 1994.

4. Golay P, Alameda L, Baumann P, Elowe J, Progin P, Polari A *et al.* Duration of untreated psychosis: Impact of the definition of treatment onset on its predictive value over three years of treatment. *Journal of Psychiatric Research* 2016; **77:** 15-21.

5. Golay P, Baumann PS, Jenni R, Do KQ, Conus P. Patients participating to neurobiological research in early psychosis: A selected subgroup? *Schizophrenia Research* 2018; **201:** 249-253.

6. Gunnar C. Topology and Data. *Bull Amer Math Soc* 2009; **46:** 255-308.

7. Bruno JL, Romano D, Mazaika P, Lightbody AA, Hazlett HC, Piven J *et al.* Longitudinal identification of clinically distinct neurophenotypes in young children with fragile X syndrome. *Proceedings of the National Academy of Sciences* 2017; **114**(40)**:** 10767-10772.

8. Lancichinetti A, Fortunato S. Consensus clustering in complex networks. *Scientific reports* 2012; **2:** 336.

9. Thiele I, Palsson BØ. A protocol for generating a high-quality genome-scale metabolic reconstruction. *Nature Protocols* 2010; **5:** 93.
