# Supplementary material for: Topology predicts long-term functional outcome in early psychosis
Source: Mol Psychiatry. 2020 Jul 6;26(9):5335–46. doi: 10.1038/s41380-020-0826-1 (PMC8589664; doi:10.1038/s41380-020-0826-1)
Supplement: Supplementary file 2 — Supplemental table 1 [file 41380_2020_826_MOESM2_ESM.pdf]

	input data		Values of c parameter																							
**model 1**	**TDA-groups**		**0.01**	**0.05**	**0.09**	**0.13**	**0.18**	**0.22**	**0.26**	**0.3**	**0.34**	**0.38**	**0.42**	**0.46**	**0.5**	**0.55**	**0.59**	**0.63**	**0.67**	**0.71**	**0.75**	**0.79**	**0.84**	**0.88**	**0.92**	**0.96**	**1**
		**A**	0.0278639	0.1484754	0.2508666	0.3333441	0.4151197	0.4679193	0.512326	0.5501835	0.5828419	0.6113072	0.6363428	0.6585371	0.6783915	0.7003661	0.7160585	0.7303234	0.7433484	0.7552895	0.7662776	0.776423	0.7880615	0.7966341	0.8046233	0.8120872	0.8190761
Training cohort cohort 1		**B**	-0.046841	-0.15706	-0.221928	-0.268146	-0.311312	-0.338322	-0.360682	-0.379549	-0.395711	-0.409726	-0.422008	-0.432865	-0.442601	-0.453313	-0.460951	-0.467887	-0.474215	-0.480012	-0.485343	-0.490263	-0.495904	-0.500058	-0.503927	-0.507541	-0.510925
Test cohort cohort 2		**C**	-0.076306	-0.250481	-0.347462	-0.413658	-0.473347	-0.509702	-0.539252	-0.563818	-0.584606	-0.602448	-0.617946	-0.631544	-0.643552	-0.65678	-0.666158	-0.674635	-0.682337	-0.689367	-0.69581	-0.701737	-0.708512	-0.713486	-0.718109	-0.722417	-0.726442
**model 2**	**k-mean clusters**		**0.01**	**0.05**	**0.09**	**0.13**	**0.18**	**0.22**	**0.26**	**0.3**	**0.34**	**0.38**	**0.42**	**0.46**	**0.5**	**0.55**	**0.59**	**0.63**	**0.67**	**0.71**	**0.75**	**0.79**	**0.84**	**0.88**	**0.92**	**0.96**	**1**
		**0_kmeans**	0.0058776	0.0665176	0.1265354	0.1755895	0.2239837	0.2549125	0.2806899	0.3024773	0.3211096	0.3372399	0.3513326	0.3637497	0.3747732	0.3869269	0.3955602	0.4033743	0.4104809	0.4169719	0.4229241	0.4284022	0.4346653	0.4392638	0.4435382	0.4475217	0.4512429
Training cohort cohort 1		**1_kmeans**	-0.030257	-0.105131	-0.151265	-0.184828	-0.216649	-0.236745	-0.253491	-0.267699	-0.279951	-0.290604	-0.299971	-0.308279	-0.3157	-0.323936	-0.329823	-0.335179	-0.340074	-0.344566	-0.348703	-0.352525	-0.356915	-0.360151	-0.363168	-0.36599	-0.368633
Test cohort cohort 2		**2_kmeans**	-0.071983	-0.233295	-0.318986	-0.374928	-0.42334	-0.451806	-0.474346	-0.492664	-0.507823	-0.520623	-0.531565	-0.541026	-0.549289	-0.558255	-0.564534	-0.570153	-0.575212	-0.579791	-0.583953	-0.587755	-0.592067	-0.595209	-0.598112	-0.600802	-0.603302
**model 3**	**clusters on PCA**		**0.01**	**0.05**	**0.09**	**0.13**	**0.18**	**0.22**	**0.26**	**0.3**	**0.34**	**0.38**	**0.42**	**0.46**	**0.5**	**0.55**	**0.59**	**0.63**	**0.67**	**0.71**	**0.75**	**0.79**	**0.84**	**0.88**	**0.92**	**0.96**	**1**
		**0_PCA**	-0.059111	-0.190106	-0.258786	-0.303209	-0.341292	-0.363472	-0.380886	-0.39492	-0.406475	-0.416137	-0.424334	-0.431371	-0.437476	-0.44405	-0.448622	-0.452689	-0.456329	-0.459605	-0.462569	-0.46523	-0.468263	-0.470462	-0.472484	-0.47435	-0.476076
Training cohort cohort 1		**1_PCA**	0.0149078	0.1018023	0.1804946	0.2438578	0.3060933	0.3458571	0.3790154	0.4070666	0.4311602	0.4519997	0.4702315	0.4863177	0.5006175	0.5164061	0.5276368	0.5378138	0.5470796	0.5555518	0.5633287	0.5704823	0.5786807	0.5847062	0.5903115	0.5955394	0.6004269
Test cohort cohort 2		**2_PCA**	-0.052548	-0.186619	-0.27016	-0.330742	-0.387792	-0.423685	-0.453513	-0.47877	-0.500536	-0.519439	-0.536057	-0.550792	-0.563956	-0.57857	-0.589022	-0.598535	-0.607234	-0.61522	-0.62258	-0.629415	-0.637237	-0.643009	-0.648395	-0.653433	-0.658157
**model 4**	**PANSS items**		**0.01**	**0.05**	**0.09**	**0.13**	**0.18**	**0.22**	**0.26**	**0.3**	**0.34**	**0.38**	**0.42**	**0.46**	**0.5**	**0.55**	**0.59**	**0.63**	**0.67**	**0.71**	**0.75**	**0.79**	**0.84**	**0.88**	**0.92**	**0.96**	**1**
		**P1**	-0.019407	-0.013298	0.010416	0.0361892	0.0683738	0.0935169	0.117986	0.1417609	0.1648557	0.187298	0.2090971	0.230324	0.250994	0.2760941	0.2956182	0.314676	0.3333276	0.3515337	0.3693417	0.3867709	0.4080512	0.4246905	0.441004	0.4570056	0.4727087
Training cohort cohort 1		**P2**	-0.02199	-0.055431	-0.076443	-0.095209	-0.117507	-0.134856	-0.151916	-0.16874	-0.185355	-0.201776	-0.218083	-0.234168	-0.250085	-0.26975	-0.285302	-0.300697	-0.315946	-0.331038	-0.345983	-0.360784	-0.379087	-0.393574	-0.407925	-0.422145	-0.436234
Test cohort cohort 2		**P3**	-0.022556	-0.045208	-0.05271	-0.057635	-0.062634	-0.066253	-0.069697	-0.073033	-0.076291	-0.079487	-0.082532	-0.085582	-0.088572	-0.092227	-0.095085	-0.097883	-0.100621	-0.103299	-0.105917	-0.108476	-0.11159	-0.114015	-0.116381	-0.118689	-0.120939
		**P4**	-0.022487	-0.054701	-0.075076	-0.094365	-0.118761	-0.138792	-0.159297	-0.180222	-0.201508	-0.223092	-0.244981	-0.267025	-0.289222	-0.317128	-0.339539	-0.361998	-0.384491	-0.406978	-0.42945	-0.451892	-0.479881	-0.502207	-0.524465	-0.546645	-0.568742
		**P5**	-0.011775	-0.007381	0.0074958	0.0231979	0.0422124	0.0566232	0.0702627	0.0831523	0.0953287	0.1068351	0.1175838	0.127826	0.1375106	0.148886	0.1574416	0.1655454	0.173259	0.1805496	0.1874662	0.1940303	0.2017701	0.2076121	0.2131614	0.2184339	0.223444
		**P6**	-0.023661	-0.027609	-0.011614	0.0074779	0.0323616	0.0523537	0.0721918	0.0917908	0.1111117	0.1301379	0.1488312	0.1672446	0.1853592	0.2075902	0.2250531	0.2422371	0.2591648	0.275817	0.2922123	0.3083575	0.3281974	0.3438039	0.3591812	0.3743353	0.389272
		**P7**	-0.019979	-0.04338	-0.0547	-0.064456	-0.076363	-0.08602	-0.09586	-0.105882	-0.116066	-0.126385	-0.136744	-0.147228	-0.157768	-0.170992	-0.181589	-0.192187	-0.202773	-0.213337	-0.223869	-0.234362	-0.247414	-0.257796	-0.268122	-0.278386	-0.288586
		**N1**	-0.037294	-0.084588	-0.10685	-0.123223	-0.139748	-0.150974	-0.160945	-0.169948	-0.17818	-0.185788	-0.192913	-0.199596	-0.205927	-0.213432	-0.219167	-0.224701	-0.230052	-0.235269	-0.240363	-0.245352	-0.251464	-0.256269	-0.261011	-0.265698	-0.270337
		**N2**	-0.036802	-0.085862	-0.109996	-0.127539	-0.144451	-0.155151	-0.163882	-0.170975	-0.17667	-0.181157	-0.184483	-0.186941	-0.188565	-0.189562	-0.189625	-0.189107	-0.188059	-0.186542	-0.184596	-0.182262	-0.178849	-0.175763	-0.172389	-0.168753	-0.164877
		**N3**	-0.026781	-0.06186	-0.079346	-0.092721	-0.106641	-0.11631	-0.12501	-0.132922	-0.140173	-0.146859	-0.153074	-0.158846	-0.164238	-0.170507	-0.175187	-0.179603	-0.183793	-0.187756	-0.191521	-0.195107	-0.199359	-0.202593	-0.205691	-0.208663	-0.211518
		**N4**	-0.042663	-0.112681	-0.157062	-0.194541	-0.235945	-0.265901	-0.293592	-0.319347	-0.343409	-0.36597	-0.387158	-0.407152	-0.426045	-0.44826	-0.465014	-0.480941	-0.496113	-0.510577	-0.524389	-0.537594	-0.553312	-0.5653	-0.576804	-0.587855	-0.59848
		**N5**	-0.030726	-0.071037	-0.0923	-0.110021	-0.130387	-0.145974	-0.161181	-0.176114	-0.19083	-0.205358	-0.219685	-0.233875	-0.247912	-0.265247	-0.278951	-0.292509	-0.305947	-0.319226	-0.332365	-0.345364	-0.361418	-0.374108	-0.386663	-0.399083	-0.411371
		**N6**	-0.038108	-0.104916	-0.150172	-0.189855	-0.235208	-0.269064	-0.301205	-0.331883	-0.361279	-0.389536	-0.416751	-0.443041	-0.468473	-0.499161	-0.522899	-0.545969	-0.568444	-0.590314	-0.611635	-0.63244	-0.657761	-0.677502	-0.696809	-0.715704	-0.734206
		**N7**	-0.024478	-0.06584	-0.09362	-0.118337	-0.147114	-0.16898	-0.190017	-0.210333	-0.23	-0.249069	-0.267466	-0.285413	-0.302858	-0.323998	-0.340406	-0.356388	-0.37197	-0.387158	-0.401973	-0.416431	-0.434021	-0.447726	-0.461115	-0.474203	-0.486999
		**G1**	-0.014787	-0.01397	-0.001115	0.0126088	0.0290252	0.0412462	0.0526274	0.0632117	0.0730569	0.0822237	0.0907143	0.0986705	0.1061024	0.1147206	0.1211243	0.1271289	0.1327768	0.1380754	0.1430585	0.1477488	0.1532307	0.1573341	0.1612048	0.1648579	0.1683076
		**G2**	-0.029653	-0.043071	-0.033562	-0.020697	-0.003677	0.0099054	0.0232433	0.0362729	0.0489786	0.0613647	0.0734755	0.0852775	0.0968086	0.1108662	0.121846	0.132604	0.1431661	0.1535214	0.1636904	0.1736826	0.1859375	0.1955628	0.2050369	0.2143664	0.2235572
		**G3**	-0.027031	-0.052506	-0.059269	-0.06271	-0.065429	-0.06703	-0.068352	-0.06949	-0.070496	-0.0714	-0.072203	-0.072949	-0.073634	-0.074414	-0.074985	-0.075512	-0.076008	-0.076463	-0.076885	-0.077277	-0.07773	-0.078064	-0.078376	-0.078667	-0.07894
		**G4**	-0.026238	-0.045885	-0.047434	-0.046721	-0.045547	-0.044932	-0.044709	-0.044881	-0.045421	-0.046291	-0.047397	-0.048793	-0.05041	-0.052696	-0.054706	-0.056854	-0.059116	-0.061484	-0.063941	-0.066473	-0.069727	-0.072391	-0.0751	-0.077846	-0.080623
		**G5**	-0.027674	-0.073608	-0.103738	-0.130107	-0.160344	-0.183009	-0.204579	-0.225201	-0.244976	-0.263982	-0.282168	-0.299768	-0.316749	-0.337166	-0.352894	-0.368116	-0.382874	-0.397171	-0.41104	-0.424502	-0.440789	-0.453407	-0.465678	-0.477617	-0.489239
		**G6**	-0.038529	-0.093592	-0.124684	-0.150223	-0.178299	-0.198701	-0.217695	-0.235512	-0.252312	-0.268215	-0.283265	-0.297624	-0.311324	-0.327605	-0.340013	-0.35192	-0.363375	-0.374388	-0.385	-0.395237	-0.407543	-0.417021	-0.426194	-0.435082	-0.4437
		**G7**	-0.029759	-0.058656	-0.066524	-0.070287	-0.072829	-0.074	-0.074742	-0.075212	-0.075507	-0.07569	-0.075835	-0.07592	-0.07599	-0.076078	-0.076162	-0.076267	-0.076402	-0.076564	-0.076758	-0.076986	-0.07732	-0.077628	-0.077971	-0.078348	-0.07876
		**G8**	-0.016117	-0.034826	-0.042914	-0.048853	-0.054961	-0.059195	-0.062973	-0.066362	-0.069406	-0.072134	-0.07437	-0.076451	-0.078259	-0.080155	-0.081397	-0.082409	-0.083194	-0.083776	-0.084159	-0.084353	-0.084343	-0.084144	-0.083784	-0.083269	-0.082608
		**G9**	-0.019776	-0.040939	-0.048937	-0.054571	-0.060435	-0.064664	-0.068646	-0.072444	-0.076098	-0.079629	-0.083104	-0.086454	-0.089724	-0.09371	-0.096826	-0.099884	-0.102885	-0.10584	-0.108749	-0.111619	-0.115153	-0.117943	-0.120702	-0.123434	-0.126139
		**G10**	-0.014548	-0.023381	-0.02113	-0.01702	-0.011159	-0.006318	-0.001485	0.0032979	0.0079477	0.0125038	0.0169422	0.0212611	0.0254612	0.0305483	0.0344918	0.0383275	0.0420687	0.0457047	0.0492464	0.0526984	0.0568932	0.0601578	0.0633454	0.0664597	0.0695041
		**G11**	-0.017397	-0.009991	0.0134324	0.0384934	0.0694071	0.0932991	0.1163446	0.1385455	0.1599357	0.1805606	0.2004945	0.2197443	0.2383723	0.2608469	0.278225	0.2951065	0.311536	0.3275212	0.3430986	0.3582927	0.3767806	0.3911921	0.4052874	0.4190835	0.4325963
		**G12**	-0.026565	-0.04818	-0.051016	-0.050889	-0.049667	-0.048482	-0.047293	-0.046154	-0.045081	-0.044074	-0.043117	-0.042214	-0.041347	-0.040301	-0.039482	-0.038672	-0.037872	-0.037062	-0.036243	-0.035413	-0.034353	-0.033484	-0.032595	-0.031684	-0.03075
		**G13**	-0.021839	-0.02877	-0.017498	-0.003062	0.0163057	0.03215	0.0480547	0.0639132	0.0796679	0.095286	0.1106995	0.1259749	0.1410735	0.1596942	0.1743885	0.1889041	0.2032388	0.2174024	0.2313949	0.2452192	0.2622685	0.2757271	0.2890287	0.3021766	0.3151743
		**G14**	-0.018974	-0.03717	-0.043164	-0.047675	-0.053147	-0.057743	-0.06258	-0.06765	-0.07292	-0.078359	-0.083827	-0.089464	-0.09517	-0.102368	-0.108153	-0.113944	-0.119725	-0.125489	-0.131224	-0.13692	-0.143978	-0.149565	-0.155096	-0.160566	-0.165971
		**G15**	-0.020901	-0.05126	-0.069059	-0.08421	-0.101462	-0.114399	-0.126754	-0.138618	-0.150049	-0.161091	-0.17182	-0.182201	-0.192285	-0.204506	-0.213998	-0.223256	-0.232308	-0.241145	-0.24979	-0.258254	-0.268597	-0.276693	-0.28464	-0.292446	-0.300118
		**G16**	-0.040273	-0.115998	-0.17024	-0.218824	-0.275048	-0.317363	-0.357728	-0.396389	-0.433527	-0.469284	-0.503781	-0.537119	-0.569386	-0.608331	-0.638459	-0.667737	-0.696237	-0.723979	-0.751019	-0.777398	-0.809497	-0.834517	-0.858985	-0.882928	-0.906372
